# Traditional Old Dietary Pattern of Castellana Grotte (Apulia) Is Associated with Healthy Outcomes

**DOI:** 10.3390/nu12103097

**Published:** 2020-10-12

**Authors:** Fabio Castellana, Roberta Zupo, Ilaria Bortone, Gianluigi Giannelli, Rossella Donghia, Luisa Lampignano, Chiara Griseta, Giovanni De Pergola, Heiner Boeing, Anna Maria Cisternino, Giancarlo Logroscino, Rodolfo Sardone, Vito Guerra

**Affiliations:** 1 Population Health Unit—“Salus in Apulia Study” National Institute of Gastroenterology “Saverio de Bellis”, Research Hospital, Castellana Grotte, 70013 Bari, Italy; zuporoberta@gmail.com (R.Z.); ilariabortone@gmail.com (I.B.); luisalampignano@gmail.com (L.L.); chiaragriseta@gmail.com (C.G.); boeing@dife.de (H.B.); rodolfo.sardone@irccsdebellis.it (R.S.); 2 Scientific Direction, National Institute of Gastroenterology “Saverio de Bellis”, Research Hospital, Castellana Grotte, 70013 Bari, Italy; gianluigi.giannelli@irccsdebellis.it; 3 Unit of Epidemiology and Biostatistics, National Institute of Gastroenterology “Saverio de Bellis”, Research Hospital, Castellana Grotte, 70013 Bari, Italy; rossydonghia@gmail.com (R.D.); vito.guerra@irccsdebellis.it (V.G.); 4 Clinical Nutrition Unit, Department of Biomedical Science and Human Oncology, School of Medicine, University of Bari, Policlinico, 70124 Bari, Italy; gdepergola@libero.it; 5 German Institute of Human Nutrition Potsdam-Rehbrücke, 14558 Nuthetal, Germany; 6 Clinical Nutrition Unit, National Institute of Gastroenterology “Saverio de Bellis”, Research Hospital, Castellana Grotte, 70013 Bari, Italy; annamaria.cisternino@irccsdebellis.it; 7 Center for Neurodegenerative Diseases and the Aging Brain, Department of Clinical Research in Neurology, University of Bari Aldo Moro, 70121 Bari, Italy; giancarlo.logroscino@gmail.com

**Keywords:** healthy diet indices, food intake, Apulia, MICOL study, greatAGE study, prospective cohort study, mind index, dash index, Meddietscore

## Abstract

Background: There is still room for further studies aimed at investigating the most widespread diets in the Mediterranean area. The objective of the study is to analyze the relation of food group intake to clinical chemical indicators of health, and also to compare the food group intake with healthy well-known diet indices. Methods: Lifestyle, dietary, and clinical data collected in 2005/2006 and 2012/2018 from Castellana Grotte, located in the rural area of Apulia, were analyzed. The study populations included newly recruited subjects at each time period (n = 1870) as well as subjects examined twice and compared over time regarding health indicators (n = 734). Diet was assessed through a validated food frequency questionnaire. Three healthy diet indices were calculated and related to 29 food groups. We also performed prospective regression of food group consumption with health indicators. Results: The diet over the time period of observation was very stable and consisted of a high proportion of vegetables, fruit and grains. No major changes in body mass index (BMI) and blood pressure were observed. Consumption of low-fat dairy, juices, olive oil, and water were related to reductions in weight gain, systolic blood pressure, high-density lipoprotein (HDL)-cholesterol and cholesterol (total and HDL) levels, in that order. Over the time periods we observed only a slight decrease of adherence to the Meddietscore. The correlations of the healthy diet indices with food groups revealed some differences among the indices, mostly regarding the intake of fruit and vegetables. Conclusions: The dietary pattern of Apulia is in line with many principles of a healthy diet and the cohort population seems to be less liable to undergo a transition to a westernized diet.

## 1. Introduction

There is still great interest in the diet practiced around the Mediterranean Sea due to the observation of low disease incidences compared to Northern European countries. A broad search for common dietary practices in this region of the world identified a relatively high proportion of energy gained from cereals, vegetables, and fruits, relatively less meat consumption, and a greater reliance on vegetables than on animal fats compared to Northern Europe [1]. These foods were recently highlighted by the Global Burden of Disease study on diet, which found that dietary factors have a high impact on disability-adjusted life years (DALYs) [2].

However, the amounts and combinations of those foods either promoting or delaying the occurrence of diseases could vary between countries and within countries between regions. Such regional variations within a country were already well documented in The Seven Countries Study from the 1960s [3], taking Italy as example [4]. In that example, diet varied manifold in key food components such as milk and cheese, vegetables, fruits, meat, egg and fish, and even sugar and sweets taking three “Seven Countries Study” areas from North to South. Such regional dietary habits have prevailed up to now and even today understanding the preventive principles associated with regional dietary habits is still a challenge. In this spirit, the publicly funded research hospital IRCCS “S. de Bellis”, located in Castellana Grotte (Apulia, Italy) started in the 1980s a population-based cohort study, and has been measuring dietary intake in this Mediterranean population since that period [5,6,7]. This interest has recently promoted the recruitment of a new cohort in order to gain a better understanding of the aging process, particularly related to neurodegeneration and functional decline [8], with overlaps with the previous study populations. This was seen as opportunity to study diet and its implication not only cross-sectionally but also longitudinally [7].

The aim of the current research was therefore to identify the existing study populations with their overlaps, to describe and analyze the dietary intake over the last two decades, and to link dietary intake longitudinally with a biomarker profile.

## 2. Methods

### 2.1. Study Populations

**Figure 1:**
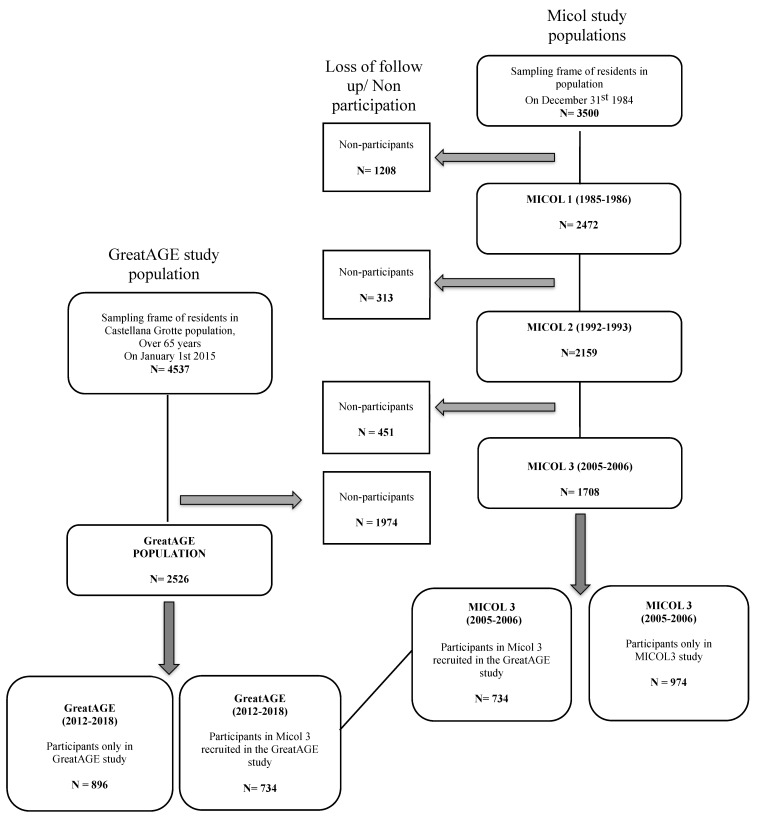
Flowchart of sample selection of Micol and GreatAGE studies. MICOL, Multicenter Italian study on Cholelithiasis.

In the beginning of the 1980s, the IRCCS de Bellis participated in the Multicenter Italian study on Cholelithiasis (MICOL) [9] and established, in 1985 for this purpose, a cohort study in Castellana Grotte, Apulia, with the aim of prospectively investigating the role of lifestyle and nutrition in gastrointestinal and other chronic diseases, among them cholelithiasis and other gallbladder diseases. Methodological details of this population-based study have previously been published [6,10]. In brief, in 1985 a random sample of 3500 subjects (2000 men and 1500 women) aged ≥30 years was drawn from the electoral roll of Castellana Grotte (17,334 residents at the 1981 Census) and invited to take part in the study. Among the 3500 invited subjects, 2472 (1429 men and 1043 women) agreed (70.6% response rate). There was no difference in age, sex, or occupation between responders and non-responders; 30% of them worked in the agricultural sector. The cohort was examined several times over the last 35 years (Figure 1). After the initial examination, the study participants were re-invited in 1992–1993 for MICOL2 (M2; 2159 participants), and in 2005/2006 for MICOL3 (MICOL3; 1708 participants). M1 included questions on the frequency of use of some food items and many other questions on lifestyle habits, M2 only some questions on culinary use in addition to the lifestyle questions, and MICOL3 included a full dietary assessment (see below in 2012, were identified as candidates for a larger population-based study conducted in the same community, the “GreatAGE” Study. The “GreatAGE” Study is an ongoing population-based cohort focusing, among other aspects, on nutrition, and age-related sensory impairments, frailty, neurodegenerative, and psychiatric diseases in the elderly [11]. In 2015, the “GreatAGE” Study was started with an invitation to the previously representative M3 participants, and in 2016, it became possible to extend the invitation to the whole 65+ population. The final study population included 2522 subjects from the 4537 originally invited. The recruitment of this study ended in 2018.

All the participants signed an informed consent document, approved by the Institutional Review Board of the National Institute of Gastroenterology and Research Hospital. For this investigation, we concentrated on the M3 and “GreatAGE” Study populations with full dietary assessments. We subdivided the M3 study population of 2005/2006 into those who joined the “GreatAGE” Study years later and those who did not or could not join the “GreatAGE” Study, mostly due to age, death, or no interest. We also subdivided the “GreatAGE” Study population of 2014/2018 into those who had already participated in the M3 examination and those who were newly recruited. By subdividing the study population, it was possible to establish a subset of study participants that was examined twice over time and, therefore, could be longitudinally compared (Figure 1). The other study participants at each point in time served as direct comparison groups to the study population examined twice.

### 2.2. Socioeconomic, Medical and Fluid Biomarker Parameters

Education was assessed by a variable having three categories (low, medium, high). Unfortunately, the variable education had to be constructed differently at each time period of examination. In the MICOL studies, educational level attained was requested in 3 categories (Primary school, secondary school, under-graduate and graduate studies). The categories were directly taken and labeled as low, medium, and high education. In the “GreatAGE” Study, the total number of years of schooling (from 0 to 18) was asked. For this study, the latter was cut in 3 categories (<5 years, 5–13, >13) that were also labeled as low, medium, and high education.

A sphygmomanometer (YTON) and a stethoscope (FARMAC-ZARBAN) were used to measure blood pressure, undertaken by professional nurses with a professional qualification in Italy. Blood pressure was determined in a sitting position after rest. The final values of blood pressure (systolic as well as diastolic blood pressure (SBP, DBP)) were the mean of the last two of three measurements. Height and weight measurements were performed using a Seca 220 altimeter and a Seca 711 scale.

Blood was collected from the subjects in the morning after an overnight fast and, among other parameters, fasting glucose (GLY), total cholesterol (TChol), high-density lipoprotein (HDL) cholesterol and triglycerides (TG) were measured, using standard automated enzymatic colorimetric methods (AutoMate 2550, Beckmann Coulter, Brea, Ca, US), under strict quality control. The prevalence of diabetes was calculated on the basis of a diagnosis of diabetes given at the interview, the use of antidiabetic medications, and fasting blood glucose above 126 (mg/dL).

### 2.3. Dietary Assessment

During the early 1990s, a better assessment of diet was needed because the 1985 29 ad hoc questions provided by the MICOL group on 4 categories of frequency of intake of specific foods and the use of olive oil and wine [6] were considered too broad. Therefore, the semi-quantitative approach of that time was followed, and a new questionnaire was developed based on foods eaten by the population. For these foods the frequency of intake of a predefined portion over the last year was probed in the questionnaire via a scale of 9 categories. Each portion was given a weight and thus the quantity of intake was calculated, expressed as mean intake in g per day. The FFQ (Food Frequency Questionnaire) was structured in 11 sections that partly mirror the sequence of foods during the day and include foods of similar characteristics: grains, meat, fish, milk and dairy products, vegetables, legumes, fruits, miscellaneous foods, water and alcoholic beverages, olive oil and other edible fats, coffee/sugar and salt. In a further step, the FFQ was validated against dietary records and the results were reviewed to make any necessary modification of the questionnaire [12].

In the final questionnaire, 85 food items were considered to best reflect the regional diet, together with some questions about the use of edible fats. The latter were not quantified, but summarized in a separate food group (19 cooking edible fats). The original FFQ has been included into the section of supplementary material.

#### 2.3.1. Food Grouping

The 85 food items of the FFQ and the questions about the use of fat were regrouped and shortened by number and also made concordant with a publication representing a Spanish (Mediterranean) diet (Appendix A (Table A1)). The publication describing the Spanish study population (controls from a nationwide case-control study on breast cancer) included 26 food groups from an original group of 117 food items [13]. For our FFQ questionnaire, we established 29 food groups of foods of similar type (Appendix A (Table A1)); 24 of them were identical to those in the Spanish publication. One food group (19 cooking edible fats) could not be quantified and was not used in this study; these foods did not contribute to nutrient intake. The establishment of 28 food groups (29 minu the 1 non−quantified food group) was also undertaken to facilitate analyses with less variables for statistical modelling and to allow direct comparisons with other studies from the Mediterranean area.

#### 2.3.2. A Priori Healthy Diet Indices

For the study, we selected three commonly used healthy diet indices, the DASH (dietary approaches to stop hypertension) diet index, the Meddietscore, and the MIND (Mediterranean-DASH intervention for neurodegenerative delay) diet index [14]. All indices had been previously shown to be related to a reduced disease risk, focusing on different aspects of the disease spectrum. The final scoring for each index had to be partly adapted to the available data regarding foods, and for the binary score (0 or 1), values the median intake of each food in the total group was used. The scoring algorithms are shown in Table 1 and the relation of the scored foods to the original foods in the questionnaire is shown in Appendix A (Table A2).

The DASH diet index goes back to a successful intervention study that reduced hypertension [15,16]. The DASH diet approach was composed of an increased consumption of fruits, vegetables, low-fat dairy, whole grains, poultry, fish, and nuts while limiting the intake of red and processed meat and added sugars. The original DASH diet was relatively low in total fat, saturated fat, and sodium [15]. The scoring of the DASH diet index in our study was based on 7 food groups and 3 dietary components (total fat, saturated fat and sodium). The DASH diet index for each subject could theoretically range from 0 (lowest) to 10 (highest concordance) (Table 1).

The concordance with the Mediterranean diet was not scored according to the Mediterranean Diet Index established by Trichopoulou et al. [17] but according to the algorithm of the Meddietscore based on the Greek Mediterranean diet pyramid with its food groups, featuring less meat and more carbohydrate-rich foods [18]. The complex scoring with up to 5 points proposed by this group was not followed but was simplified to a binary score of 0 and 1 depending on the quantity of intake (Table 1). The Meddietscore in this study included 11 dietary components, each scored either as 0 or 1, yielding a score of maximum 11 points. Details of the dietary components are shown in Table 1 and the concordance between the original foods and the Med diet components can be found in additional file 1 (Table A2).

The MIND diet index by Morris et al. [14] is associated with a reduction in cognitive decline. The MIND diet index includes 15 dietary components, composed of 10 brain-healthy food groups (green leafy vegetables, other vegetables, nuts, berries, beans, whole grains, fish, poultry, olive oil and wine) and 5 brain-unhealthy food groups (red meats, butter and margarine, cheese, pastries and sweets, and fried/fast food). For the diet score components of the MIND diet index, we assigned either a score value of 0 or 1 according to the median intake (Table 1). The MIND diet score ranged between 0 and 15 (Table 1) and the concordance between the original foods in the questionnaire and the Mind-diet components can be found in additional file 1 (Table A2).

### 2.4. Statistical Analysis

Data were reported as means ± standard deviations (X ± SD) for continuous measures, and frequency and percentages (%) for all categorical variables, separated according to the study populations (Table 2). For Table 3, food intake (g/day) of each study population was standardized to an energy intake of 2000 Kcal/day for description and comparison purposes. The standardization was done by dividing the total daily individual food quantity in grams/day by Kcal/day, and then multiplying with 2000. The relation between food group intake and health indicators was evaluated prospectively. The food group intake at MICOL3 was regressed to health indicators at “GreatAGE” (n = 734). For this analysis, specific subgroups were formed, free of impaired health indicators at the MICOL3 examination (body mass index (BMI): subjects with a BMI gain higher than 1.5 (n = 605 remained); SBP: No increased systolic blood pressure (n = 373 remained); DBP: No increased diastolic blood pressure (n = 373 remained); GLYCEMIA: subjects with a fasting blood glucose above 126 mg/dL, without a diagnosis of diabetes and use of diabetes medications (n = 592 remained); TChol, HDL: subjects without hypercholesterolemia (<200 mg) and use of statins (n = 310 remained); TG: subjects without hypertriglyceridemia (150 mg) and use of statins (n = 458 remained). The final numbers were further reduced due to missing information in the covariates (see Table 4)). The prospective relation was investigated via a regression model that included covariates (see legend Table 4). It should be noted that the regression analyses were run with models either including or excluding the other food groups. The fat to carbohydrate ratio included, as the fat component, the intake of lipids taken from the nutrient table and, as the carbohydrate component. A *p* value < 0.05 was considered as significant.

## 3. Results

The characteristics of the study populations are shown in Table 2. In 2005/2006 the MICOL3 participants were about 65 years old, with a slightly smaller percentage of women than men. There were no particular differences between the two study populations of the MICOL3 examination in 2005/2006. Likewise, the two “GreatAGE”-Study populations, which were aged 73.5 years on average at study examination in 2012/2018, did not show particular differences in regard to anthropometry, and other health indicators. When comparing the two time periods of examination (2005/2006 vs. 2012/2018), not much change could be observed. The difference over time seen in the education variable based on methodological differences (see method section). Of particular interest is the lack of an overall decrease in health conditions referred to the MICOL3/GreatAGE study population as well as the other population groups which served as control samples, not being affected by repeated study participation. Weight gain and a substantial increase in blood pressure did not occur within the time period and indeed, as regards the clinical chemical health indicators, some improvements could actually be observed. This was true in particular of cholesterol in blood. There was also a remarkable stability of energy intake among the study populations and over time, allowing a combined dietary analysis across all study populations. Of importance in the long run could be the observation of an increased fat component compared to the carbohydrate component observable in the fat/carbohydrate ratio, which rose from 0.18 to 0.22.

The dietary data, standardized to 2000 kcal, also showed stability regarding the intake of specific foods such as fruits, and other important foods characterizing the Mediterranean diet (Table 3). Over the two time periods, the mean intake of root and other vegetables increased, as well as of olive oil, and water when taking the mean intake measurements of all 4 study groups and allowing a minimal difference of 3 g/day between the groups at each point in time (Table 3). Likewise, the intake of grains (pasta and bread) and beer decreased. Red meat was partly replaced by white meat.

Next, the role of food intake per health indicators, as shown in Table 1, was prospectively investigated. The intake of the 29 food groups in 2005/2006 was examined in relation to the health indicators in 2012/2018 by forming subgroups without impaired health indicators at the 2005/2006 examination (see method section). In Table 4, the results from the regression models are shown as p-values and the direction of relations. BMI gain (>1.5 units) between the two time periods and the values of SBP, DBP, GLY, total and HDL cholesterol, and TG in 2012/2018 were investigated for their relation with food group intake in 2005/2006 by calculating two models, one without adjustment for other food groups and one with adjustment for all other food groups. Overall, each regression model with a biomarker as dependent variable at the second examination was adjusted for gender, age, educational score, smoking, BMI, and value of the dependent variable at the first examination, and medication at the second examination (for details of the models see legend Table 4). Ideally, both analyses should come to the same conclusion, which was primarily the case for the many non-significant results. Significant results with agreement between the two models regarded low fat dairy and reduced weight gain, juices and reduced SBP, olive oil and reduced HDL-cholesterol, and water and reduced cholesterol (total and HDL) levels. Next, the significant results without adjustments for other food groups which lost significance after adjustment for other foods are described. We found an inverse relation between seafood/shellfish and total cholesterol and a positive relation between sugary foods and HDL-cholesterol. Finally, we like to mention the relations that became significant after adjustment for other foods. For most health indicators, one or two food groups subsumed all the variance of the food groups and became significant, with the exception of the health indicator DBP. We found a direct relation between leafy vegetables and an inverse relation between other vegetables and SBP, an inverse relation of the same food group with glucose, a direct relation between sugar and an inverse relation between coffee and total cholesterol, and increased triglycerides with eggs and red meat, but decreased triglycerides with ready-to-eat dishes.

Next, we investigated the healthy diet indices. The indices were constructed with the medians for the total group, allowing analysis of changes over time and between groups. The DASH diet index score in 2005/2006 was 5.3 and 5.1, (MICOL3/GreatAGE and MICOL3 only) and in 2012/2018 it was 5.1 and 4.9 (MICOL3/GreatAGE and GreatAGE only); the Meddietscore in 2005/2006 was 5.7 and 5.5, and in 2012/2018 it was 5.2 and 5.3; and the MIND diet index in 2005/2006 was 7.4 and 6.8, and in 2012/2018 it was 6.7 and 6.6, taking into account the 4 subgroups. Thus, we could observe a slightly decreased adherence to the Meddietscore between the two time periods, and to a lesser extent also for the MIND diet index. None of the indices showed a significant relation to the health indicators (data not shown).

**Figure 2:**
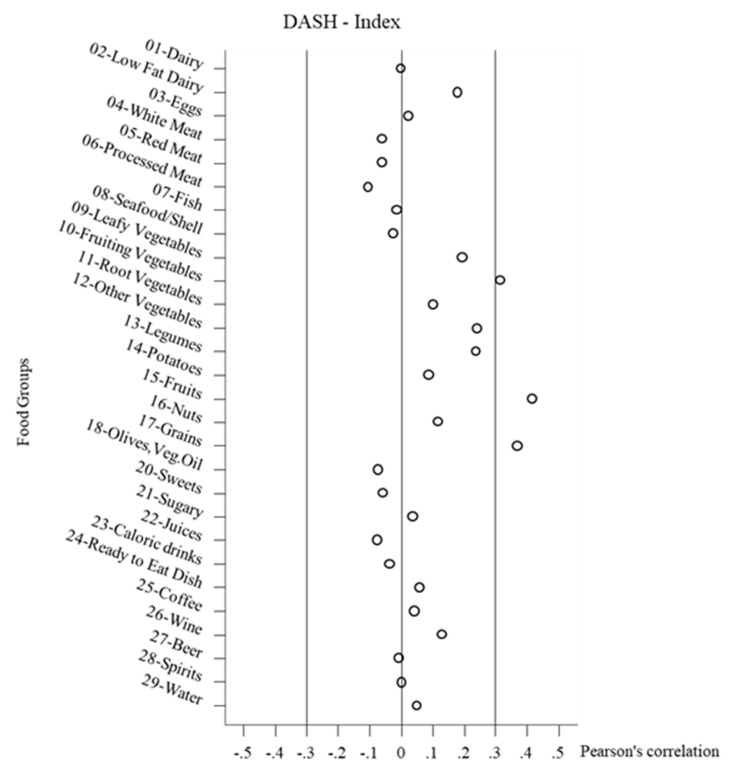
Pearson’s correlation between food groups intake and DASH-Index. DASH, dietary approaches to stop hypertension.

**Figure 3:**
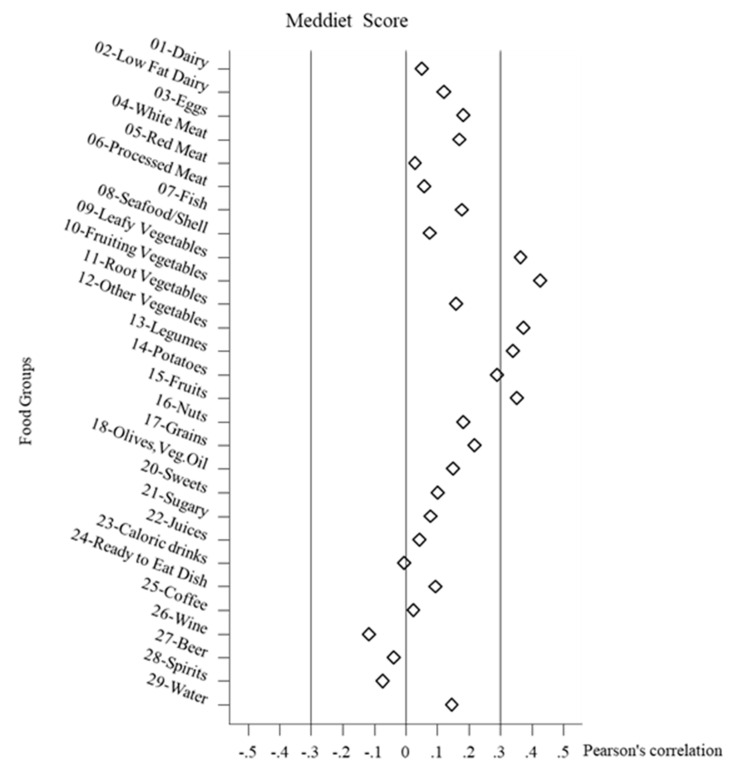
Pearson’s correlation between food groups intake and Meddietscore.

**Figure 4:**
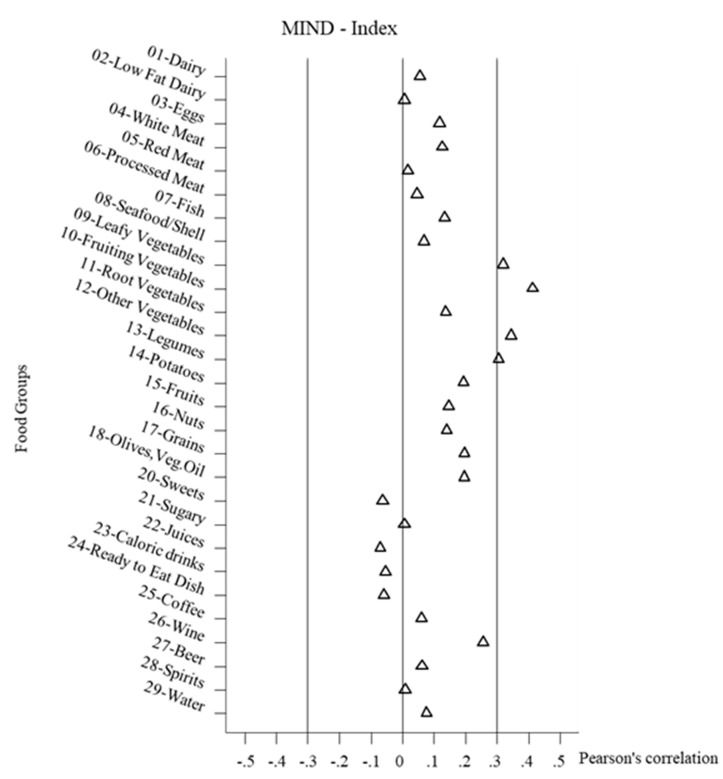
Pearson’s correlation between food groups intake and MIND-Index. MIND, Mediterranean-DASH intervention for neurodegenerative delay.

Finally, in Figure 2, Figure 3 and Figure 4, the correlations between the healthy diet indices and the food groups are shown, combined across all the study populations Overall, the correlations between the food groups and the indices were not very strong, but often several food groups stood out. Negative correlations did not reach a value < −0.2. It must be noted that foods could be correlated with the indices without these foods necessarily forming the index. For the DASH-diet index the leading positive correlations (>0.3) were with fruiting vegetables, fruits, and grains. For the Meddietscore the leading positive correlations (>0.3) were with leafy vegetables, fruiting vegetables, other vegetables, legumes, fruit, and borderline potatoes. For the MIND diet index, the leading positive correlations (>0.3) were with leafy vegetables, fruiting vegetables, other vegetables, and borderline legumes. As regards beverages, only the positive relation between wine and the DASH index stood out, whereas the other two indices were not well correlated with beverages. It could also be observed that the diet healthy indices scores were mostly independent of the intake of sweet food groups. In terms of dairy products, weak correlations were found, with the exception of a positive relation between the DASH diet index and low fat dairy. The DASH diet index also differs from the other indices in terms of the slight negative correlations with meat food groups.

## 4. Discussion

This study evaluated the dietary intake of the population of Castellana Grotte, located in a rural area in Apulia, at two different time periods and related the dietary intake to health indicators and healthy diet indices. Within the observation period of 7 years from 2005/2006 to 2012/2018, covering the lifetime age periods between 65 to 73 years on average, the source population studied showed a remarkable stability in terms of dietary intake of the 29 food groups considered, and in terms of health indicators such as anthropometry, blood pressure and clinical biochemistry. Only the intake of bread and pasta (grains) was partly replaced by olive oil, slightly increasing the fat-to-carbohydrate ratio. It could also be seen that dietary intake related prospectively to health indicators, such as olive oil that was inversely correlated to HDL-cholesterol. Three established healthy diet indices were constructed and correlated with food group intake, showing that the DASH-index represents particular fruit and grain intake and the MIND-index vegetable intake.

The economy of Castellana Grotte and the surrounding area is based on agricultural production, mainly olives, grapes, cherries, and cattle. The local diet makes use of the existing agricultural infrastructure and, for example, did not include a lot of fish and seafood, available 15 km away at the coastal sites around Monopoli. The local production of most of the food products eaten, in addition to the dietary tradition of a small town, resulted in a remarkable stability of dietary habits, characterized by high intakes of vegetables, fruit, and local pasta. The slightly lower energy intake of the older subgroups is in line with the biologically reduced energy requirement due to the reduced physical activity and muscle mass [19]. The study also showed that the population with repeated examinations in the study did not differ from the study populations only invited once, indicating a negligible influence of a healthy participant bias in terms of the dietary estimates. Whereas vegetables and fruits are established characteristics of the Mediterranean diet, the Apulian diet differs from that of other regions around the Mediterranean Sea in regard to the type of grain products. Local grain products are based on semolina meal, which is richer in dietary fiber, vitamins of the B-complex, and minerals, as compared to the flours used in industrial products [20], especially if a brown, non-refined version of Semolina flour has been used. A typical bread is called “Altamura bread”, first made in Altamura, a city near Bari and Castellana. It is obtained from milled durum wheat semolina from the Alta Murgia in the Province of Bari, produced with a natural sourdough. In 2003, “Altamura bread” was recognized as DOP (Protected Designation of Origin) by the European Community. Semolina meal is not only used for bread produced locally by small enterprises, or homemade, but more importantly, for fresh pasta (Orecchiette), which constitutes a traditional typical food product accompanied by vegetable consumption.

There is continuous debate as to whether local dietary habits will survive the next decades. The current study results offer a more positive view on this issue. We show that this elderly population has followed stable dietary habits over the last decade. Recently, it was stated that the intake of cereals (including pasta, bread and similar), whole grain bread, vegetables, legumes and fish significantly increased between 1985–1986 and 2005–2006 in this study population, whilst the consumption of fruits, meat, poultry, dairy, olive oil and alcohol significantly decreased [6]. The most dramatic decrease was observed for olive oil, that declined by 2.35 points in younger people and by 0.89 in older age groups [6]. These findings between 1985 and 2005/2006 are based on a broader type of dietary assessment [7]. Currently, these trends seem to be partially reversed, in particular in regard to olive oil and vegetables. However, our study did not address a younger population, which seems to be more sensitive to dietary changes, as the study by Veronese et al. showed. The decreased intake of red meat is in line with the regional trends of consumption, using data from the National Food Consumption Survey from 2005–2006 [21]. Our study could confirm the finding by Veronese et al. [6] that over time, adherence to the Mediterranean diet in general is slightly decreasing. However, in terms of dietary change it should be borne in mind that in Southern Italy, adherence to the Mediterranean Diet also depends on the general economic situation, as exemplified by the 2007/2010 crisis [22].

The stable consumption of vegetables and fruit could be one of the reasons why the study populations as a whole did not show increases in BMI, and also only a small increase in blood pressure within the observation period, despite being in a critical age class for the deterioration of health indicators. Of further note is the slight decrease of basic clinical chemistry parameters, such as cholesterol and fasting glucose, along with weight stability. The laboratory which analyzed the samples is also involved in clinical practice and performs continuous quality control, which should exclude such a drift due to methodological changes. The only explanation could be the increased use of medication, such as statins, that improve the clinical chemical profile. We found that about one fifth of the subjects were taking this type of medication, confirming the overall trend of high use in Italy [23].

In regard to the prospective relation between food intake and health indicators, we would like firstly to discuss those associations which showed consistency between the statistical models. These regard low fat dairy, juices, olive oil, and water. The finding of a direct association between low-fat dairy products but not high fat dairy consumption and weight gain (BMI > 1.5) in about 7 years is not supported by findings in the literature [24], and is thus a surprising result. We can only consider the role of fat in the discussion about obesity, and messages to circumvent this relation by eating low-fat products. Thus, our finding could be indicative for populations exposed to such concepts. However, low-fat products are not low energy products, since most of them provide similar energy to dairy products with regular fat, as was recognized more than 10 years ago [25]. It might not be surprising that subjects concerned about weight gain would start eating low-fat dairies without considering the energy provided by such products. More support from the literature is available for the finding that juices were associated with lower systolic blood pressure. In a recent meta-analysis of observational studies, Zheng et al. came to the same conclusion and discussed the biological mechanism underlying this finding [26]. Also the finding regarding olive oil and an inverse association with HDL-cholesterol is supported by a recent meta-analysis of intervention studies [27]. The authors of this meta-analysis pointed out that the effect is only seen with virgin olive oil, rich in phenols. The population in Castellana mainly consumes this type of olive oil because it is produced locally. Also, the inverse association between water and total HDL-cholesterol is supported by the only (to the best of our knowledge) intervention study in which water with a high content of minerals reduced LDL and HDL-cholesterol [28].

For the other relationships, some doubts arise as to the significance of the statistical behavior. If the initial relation disappears after adjustment, we need to assume that statistical interferences of other foods have taken place. If significance only appeared after adjustment for other foods it is not obvious why. For the statistical findings with no agreements between the models, the interrelations among food intake play an important role firstly attributable to statistical properties and only secondly to biological mechanisms. Therefore, we discuss here only some results from the final regression models adjusted for other foods which are widely discussed in the literature. For triglycerides, we observed a direct relation with eggs and red meat, and an inverse relation with ready-to-eat dishes. The first two findings were not supported by meta-analyses of intervention studies [29,30]. The finding regarding eggs is also in contrast to a recent publication from Spain that found the contrary [31]. For the second finding, ready-to-eat dishes could contain fats that decrease triglycerides if eaten at the expense of carbohydrates [32]. Finally, we would like to draw attention to coffee, which is consumed in this area mostly as expresso. The Dutch food-based dietary guidelines recommend not to use unfiltered coffee due to intervention studies showing an increase in cholesterol compared to filtered coffee [33]. In Apulia, as in all Italy, coffee is produced by expresso machines which do not use a filter. Fortunately, we did not observe any cholesterol increase with increasing consumption of coffee; indeed, the contrary was observed when considering the fully adjusted model.

The correlations between complex healthy diet indices and single food groups could help to understand the significance of the indices themselves in the context of the overall diet and of diet-health associations. Indices are currently widely used in the nutritional epidemiological literature due to the conviction that health effects are best addressed by a number of dietary principles [34,35]. However, indices are based only on a proportion of the dietary information for scoring, and it is of interest to know the whole picture when using such indices in studies.

It was not surprising that the indices were correlated with the intake of fruit and vegetables, with some differences in number and type of food group, in accordance with the general principles of a healthy diet. Nevertheless, the correlation analyses show that each of the indices has a slightly different significance in terms of overall food group intake. At this stage of the analysis and in view of the low to moderate correlations, it would be premature to predict the performance of the indices regarding disease outcomes with different dietary hypotheses. However, we can now be sure that the application of the indices in statistical risk analyses is not guided by side effects of other foods, not being part of the scoring algorithm.

In this context, the study by Castello et al. [13] in Spain is interesting because they used a similar approach, relating food group intake to dietary health indices. Their results regarding the aMEDindex (Alternative Mediterranean Diet Index) can be directly compared to our results regarding the Meddietscore. There seems to be little difference in the overall profile between their study and ours, demonstrating the validity of the Mediterranean diet concept across regions.

The strengths of our investigation are the large number of subjects, the concurrent investigation of a control group for the group of subjects with the two measurements, and the use of a validated dietary questionnaire. A further advantage of the study is the high participation rate at the start of the cohort and at the different subsequent examinations. Despite the losses to follow-up at each examination time, we can assume that the population was still representative of the local population at each time period. The internal validity of the data can, therefore, be considered high, since—at each time period—the subgroups did not show large differences. Nevertheless, we admit that the questionnaire was not designed to capture, in particular, changes of diet. Like many others, we consider the FFQ more as a ranking tool with less focus on the true quantitative intakes, owing to the use of a fixed number of foods and an often arbitrary definition of the portion sizes.

## 5. Conclusions

In conclusion, the study showed that the Apulian diet includes many aspects of a healthy diet. The dietary intake does not show a rapid transition towards a westernized diet, composed of a high proportion of animal foods including meat and only a small proportion of vegetables and fruit. At first glance, the diet of the Castellana Grotte region seems to be positively associated with health indicators and probably health. However, the analyses also showed that diet is a complex issue, even when condensed to 29 food groups and three healthy diet indices.

## Figures and Tables

**Table 1:** The scoring algorithms of the DASH diet score, Meddietscore and MIND diet score.

DASH Diet Score		Meddiet Score		MIND Diet Score	
DASH Components		Med Diet Components		Mind Diet Components	
Frequencies Or Specified	Max score	Frequencies Or Specified	Max score	Frequencies Or Specified	Max score
Total Grains ≥ Median	1	Non Refined Grains > Median	1	Whole Grains ≥ Median	1
Vegetables ≥ Median	1	Vegetables > Median	1	Green Leafy ≥ Median	1
Fruits ≥ Median	1	Potatoes > Median	1	Other Vegetables ≥ Median	1
Dairy ≥ Median	1	Fruits > Median	1	Berries ≥ Median	1
Meat, Poultry And Fish ≤ Median	1	Full-Fat Dairy ≤ Median	1	Red Meats And Products < Median	1
Nuts, Seeds and Legumes ≥ Median	1	Red Meat ≤ Median	1	Fish ≥ Median	1
Total Fat ≤ Median	1	Fish > Median	1	Poultry ≥ Median	1
Saturated Fat ≤ Median	1	Poultry ≤ Median	1	Beans > Median	1
Sweets ≤ Median	1	Legumes, Nuts and Beans > Median	1	Nuts ≥ Median	1
Sodium ≤ Median	1	Olive Oil ≥ Median	1	Fast/Fried Food < Median	1
		Alcohol < Median and Median > 0	1	Olive Oil Primary Oil > Median	1
				Cheese < Median	1
				Pastries, Sweets < Median	1
				Alcohol/Wine < Median	1
				Butter, Margarine < Median	1
Total Dash Diet Score	10	Total Meddietscore	11	Total Mind Diet Score	15

DASH, dietary approaches to stop hypertension; MIND, Mediterranean-DASH intervention for neurodegenerative delay.

**Table 2:** Characteristics of the study population.

	MICOL3 Examination (2005–06)		GreatAGE Study Examination (2012–2018)	
Variables *	MICOL3/GreatAGE Study Population First Assessment (n = 734)	Other MICOL3 Study Participants (n = 974)	MICOL3/GreatAGE Study Population Second Assessment (n = 734)	Other GreatAGE Study Participants (n = 896)
Sex, Women (%)	322 (42.20)	449 (46.10)	322 (42.20)	445 (49.72)
Age (years)	65.95 ± 6.59	65.05 ± 11.28	73.54 ± 6.61	73.30 ± 6.37
BMI (kg/m^2^)	29.83 ± 5.04	29.66 ± 5.47	29.09 ± 5.15	29.04 ± 4.96
Pressure (mmHg)				
Systolic	132.10 ± 19.12	129.69 ± 19.22	133.32 ±14.87	132.85 ± 14.11
Diastolic	75.63 ± 9.39	75.66 ± 9.83	78.60 ± 8.23	78.38 ± 7.88
Prevalent Diabetes ^§^ (%)	112 (15.26)	178 (18.37)	109 (14.87)	109 (12.98)
Total Cholesterol (mg/dL)	202.26 ± 37.47	198.51 ± 41.42	183.68 ± 38.53	173.72 ± 55.28
HDL Cholesterol (mg/dL)	51.18 ± 13.92	50.82 ± 13.08	48.91 ± 13.07	46.05 ± 18.25
Triglycerides (mg/dL)	135.68 ± 95.40	143.16 ± 100.59	108.77 ± 61.74	96.42 ± 57.99
Glucose (mg/dL)	112.14 ± 32.67	113.55 ± 33.56	107.49 ± 30.45	105.66 ± 26.21
Energy Intake (Kcal)	2182.76 ± 773.06	2187.82 ± 858.04	2060.15 ± 805.61	2015.13 ± 803.37
Fat/Carbohydrate Ratio	0.18 ± 0.09	0.17 ± 0.08	0.22 ± 0.25	0.21 ± 0.13

* Reported as: Mean and Standard Deviation (X ± SD); ^§^ Diabetic, Glycemia ≥ 126 (mg/dL). Abbreviations: Number (n), Body Mass Index (BMI).

**Table 3:** Food group intake within the study populations at the two examination periods (MICOL3 (Multicenter Italian study on Cholelithiasis) and GreatAGE study).

	MICOL3 (2005–06)		GreatAGE Study (2012–18)	
Food Groups *	Micol3/GreatAGE Study Population First Assessment (n = 734)	Other Micol3 Study Participants (n = 974)	Micol3/GreatAGE Study Population Second Assessment (n = 734)	Other GreatAGE Study Participants (n = 896)
Dairy	89.18 ± 82.14	101.56 ± 96.64	100.54 ± 99.43	104.12 ± 95.22
Low Fat Dairy	97.33 ± 112.75	107.55 ± 127.61	104.98 ± 116.78	106.67 ± 113.30
Eggs	7.02 ± 7.54	7.18 ± 8.43	7.32 ± 7.81	9.07 ± 9.26
White Meat	22.05 ± 19.70	22.72 ± 22.70	25.80 ± 25.53	28.88 ± 51.82
Red Meat	28.91 ± 23.63	28.51 ± 22.10	24.81 ± 20.00	22.39 ± 21.48
Processed Meat	14.44 ± 13.22	15.73 ± 14.04	14.44 ± 12.42	15.49 ± 13.77
Fish	25.01 ± 23.76	24.96 ± 21.12	26.16 ± 23.24	27.99 ± 28.80
Seafood/Shellfish	10.80 ± 10.98	9.88 ± 10.20	9.98 ± 14.84	10.17 ± 15.43
Leafy Vegetables	63.14 ± 60.53	58.00 ± 68.05	59.44 ± 62.17	62.74 ± 60.47
Fruiting Vegetables	93.24 ± 74.51	81.70 ± 61.23	91.82 ± 72.09	95.51 ± 71.02
Root Vegetables	7.81 ± 20.72	7.94 ± 15.04	11.00 ± 23.10	13.05 ± 30.16
Other Vegetables	74.65 ± 64.43	66.52 ± 71.46	82.31 ± 74.93	80.71 ± 69.74
Legumes	37.35 ± 25.15	34.60 ± 23.23	38.12 ± 23.47	40.04 ± 31.45
Potatoes	14.02 ± 13.88	14.48 ± 17.99	13.65 ± 17.30	12.98 ± 14.95
Fruits	595.53 ± 405.44	603.18 ± 410.51	584.29 ± 405.42	594.17 ± 412.87

All statistical analyses were performed using Stata software, version 15.1 (Statacorp LP, Lakeway Drive, TX, USA). * Standardized for energy intake (2000 Kcal). Abbreviations: Number (n). All food groups were calculated on daily consumption (g/day).

**Table 4:** *p*-values of linear regression modelling the relation between intake of food groups in MICOL3 and health indicators at “GreatAGE”.

	Health Indicators													
	BMI Gain > 1.5	DBP		SBP		GLY		TChol		HDL-Chol		TG		
	(kg/m^2^) (n = 114)	(mmHg) (n = 350)		(mmHg) (n = 350)		(mg/dL) (n = 573)		(mg/ dL) (n = 232)		(mg/ dL) (n = 231)		(mg/ dL) (n = 336)		
*FOOD GROUP*	*p* value of Semi ^+1^ adjusted model	*p* value of the fully * Adjusted model	*p* value of Semi ^+2^ adjusted model	*p* value of the fully * Adjusted model	*p* value of Semi ^+3^ adjusted model	*p* value of the fully * Adjusted model	*p* value of Semi ^+4^ adjusted model	*p* value of the fully * Adjusted model	*p* value of Semi ^+5^ adjusted model	*p* value of the fully * Adjusted model	*p* value of Semi ^+6^ adjusted model	*p* value of the fully * Adjusted model	*p* value of Semi ^+7^ adjusted model	*p* value of the fully * Adjusted model
*Dairy*	0.920	0.946	0.489	0.643	0.335	0.244	0.849	0.960	0.718	0.547	0.791	0.928	0.894	0.829
*Low Fat Dairy*	**↑0.035**	**↑0.003**	0.350	0.490	0.380	0.284	0.773	0.723	0.058	0.153	0.432	0.382	0.933	0.689
*Eggs*	0.751	0.617	0.634	0.750	0.588	0.720	0.763	0.777	0.222	0.341	0.125	0.470	0.074	**↑0.046**
*White Meat*	0.254	0.644	0.526	0.591	0.816	0.462	0.277	0.780	0.902	0.811	0.487	0.875	0.739	0.502
*Red Meat*	0.958	0.341	0.536	0.323	0.380	0.180	0.891	0.843	0.535	0.972	0.493	0.581	0.071	**↑0.046**
*Processed Meat*	0.102	0.245	0.683	0.66	0.401	0.482	0.700	0.996	0.271	0.519	0.172	0.39	0.526	0.813
*Fish*	0.205	0.125	0.624	0.443	0.966	0.751	0.21	0.21	0.059	0.588	0.838	0.787	0.108	0.186
*Seafood/Shellfish*	0.600	0.823	0.951	0.438	0.956	0.835	0.833	0.189	**↓0.026**	0.252	0.626	0.868	0.539	0.763
*Leafy Vegetables*	0.733	0.171	0.866	0.708	0.305	**↑0.041**	0.492	0.796	0.687	0.299	0.754	0.464	0.608	0.958
*Fruiting Vegetables*	0.157	0.094	0.419	0.741	0.958	0.553	0.902	0.177	0.058	0.354	0.696	0.774	0.111	0.171
*Root Vegetables*	0.566	0.613	0.811	0.972	0.811	0.300	0.548	0.801	0.722	0.602	0.697	0.957	0.273	0.685
*Other Vegetables*	0.399	0.570	0.274	0.467	0.632	**↓0.049**	0.082	**↓0.018**	0.174	0.288	0.803	0.986	0.209	0.431
*Legumes*	0.675	0.64	0.713	0.744	0.705	0.765	0.148	0.339	0.547	0.266	0.929	0.664	0.361	0.700
*Potatoes*	0.389	0.522	0.32	0.483	0.855	0.712	0.934	0.690	0.259	0.184	0.396	0.367	0.484	0.954
*Fruits*	0.791	0.968	0.480	0.177	0.719	0.363	0.569	0.533	0.466	0.627	0.859	0.337	0.501	0.159
*Nuts*	0.564	0.783	0.807	0.638	0.142	0.190	0.996	0.877	0.901	0.374	0.517	0.529	0.960	0.791
*Grains*	0.909	0.909	0.623	0.696	0.923	0.935	0.873	0.982	0.740	0.342	0.307	0.116	0.208	0.436
*Olives And Vegetable Oil*	0.680	0.203	0.540	0.470	0.447	0.388	0.132	0.074	0.260	0.635	**↑0.002**	**↑0.001**	0.706	0.347
*Sweets*	0.325	0.829	0.795	0.808	0.142	0.264	0.357	0.256	0.343	0.431	0.120	0.085	0.629	0.718
*Sugary*	0.190	0.584	0.964	0.877	0.867	0.867	0.056	0.196	0.133	**↑0.046**	**↑0.039**	0.137	0.785	0.968
Juices	0.633	0.594	0.785	0.766	**↓0.036**	**↓0.009**	0.371	0.281	0.207	0.197	0.984	0.930	0.689	0.594
*Caloric Drinks*	0.779	0.360	0.954	0.883	0.404	0.657	0.536	0.646	0.336	0.955	0.382	0.888	0.754	0.804
*Ready To Eat Dish*	0.574	0.815	0.612	0.667	0.813	0.958	0.247	0.339	0.121	0.406	0.845	0.836	0.244	**↓0.021**
*Coffee*	0.673	0.416	0.957	0.982	0.969	0.871	0.067	0.126	0.149	**↓0.010**	0.075	0.235	0.591	0.445
*Wine*	0.767	0.599	0.281	0.192	0.556	0.537	0.121	**↓0.040**	0.973	0.922	0.822	0.112	0.675	0.602
*Beer*	0.097	0.095	0.995	0.960	0.856	0.719	0.308	0.258	0.224	0.153	0.372	0.424	0.820	0.699
*Spirits*	0.857	0.595	0.912	0.670	0.958	0.652	0.742	0.319	0.931	0.492	0.148	0.063	0.866	0.638
*Water*	0.772	0.510	0.62	0.885	0.456	0.218	0.815	0.927	**↓0.028**	**↓0.008**	**↓0.005**	**↓0.005**	0.899	0.717

Abbreviations: SBP: Systolic blood pressure; DBP: Diastolic blood pressure; GLY: fasting glucose; TChol: total cholesterol; HDL-Chol: high-density lipoprotein cholesterol; TG: Triglycerides. ^+^ Modelling. Semi-adjusted models. ^1^ Body mass index (BMI) delta adjusted for: SEX, AGE, YEARS OF EDUCATION, SMOKING. ^2^ SBP adjusted for: SEX, AGE, YEARS OF EDUCATION, SMOKING, BMI, SBP at M3, MEDICATION FOR HYPERTENSION AT GA. ^3^ DBP adjusted for: SEX, AGE, YEARS OF EDUCATION, SMOKING, BMI, DBP at M3, MEDICATION FOR HYPERTENSION AT GA. ^4^ Total cholesterol level adjusted for: SEX, AGE, YEARS OF EDUCATION, SMOKING, BMI, TOTAL CHOLESTEROL at M3, MEDICATION FOR DYSLIPIDAEMIA AT GA. ^5^ HDL cholesterol level adjusted for: SEX, AGE, YEARS OF EDUCATION, SMOKING, BMI, HDL CHOLESTEROL, MEDICATION FOR DYSLIPIDAEMIA AT GA. ^6^ Triglycerides level adjusted for: SEX, AGE, YEARS OF EDUCATION, SMOKING, BMI, TRIGLYCERIDES at M3, MEDICATION FOR DYSLIPIDAEMIA AT GA. ^7^ Glucose blood level adjusted for: SEX, AGE, YEARS OF EDUCATION, SMOKING, BMI, GLUCOSE BLOOD LEVEL at M3, MEDICATION FOR DIABETES AT GA. * Fully adjusted model (semi adjusted models plus all food groups).

## Supplementary Materials

The following are available online at https://www.mdpi.com/2072-6643/12/10/3097/s1.

## Appendix A

Traditional old dietary pattern of Castellana Grotte (Apulia) is associated with healthy outcomes.

**Table A1:** Concordance of the single food in the questionnaire and the food grouping used in the analyses.

Final Food Groups	Single Foods from Questionnaire
DAIRY	Latte intero (*whole-fat milk*), Scamorza-Caciottina fresca-Stracchino-Fontina (semi-seasoned italian cheese), Bel Paese-Gorgonzola (italian blue cheese), Provolone-Caciocavallo (seasoned italian cheese), Grana-Parmigiano, Svizzero (*swiss cheese*), Pecorino-Vacchino (*goat cheese, cow cheese*), Formaggino, (*cheese spread*), Mozzarella (*mozzarella cheese*), Gelato (*ice cream*), Yogurt
LOW FAT DAIRY	Latte scremato–parzialmente scremato (*skimmed and semi-skimmed milk*), Ricotta (*cottage cheese*)
EGGS	*Eggs*
WHITE MEAT	*Chicken*, *Rabbit*
RED MEAT	Vitello (*veal*), Cavallo (*horse*), Maiale (*Pork*), Fegato (*liver*), Agnello (*lamb*)
PROCESSED MEAT	Salsiccia fresca (*fresh sausages*), Prosciutto crudo (*raw ham*), Mortadella (a tipical italian cured meat),Prosciutto cotto (ham), Salame (*salami*)
FISH	Sogliola-Orata-Dentice-Spigola-Cernia (*sole, sea bream, snapper, sea bass, grouper*), Merluzzo-Razza-Palombo (*codfish, stingray, dogfish*), Triglia-Cefalo-Sgombro (*goatfish, mullet, mackerel*), Acciughe-Sarde (*anchovies, sardines*), Tonno sott’olio (*tuna in oil*)
SEAFOOD/SHELLFISH	Polpo-Seppie-Calamari-Gamberi (*octopus, cuttlefish, squid, prawns)*, Cozze-Altri frutti di mare (*mussels, other seafoods*)
LEAFY VEGETABLES	Spinaci (*spinach*), Bietole-Cicorie (*chard, chicory*), Insalata (*salad*)
FRUITING VEGETABLES	Pomodori (*tomatoes*), Zucchine-Melanzane (*zucchini*, *eggplants*), Peperoni (*peppers*), Carciofi (*artichokes*) Cetrioli-cocomeri (*cucumbers*)
ROOT VEGETABLES	*Carrots*
OTHER VEGETABLES	Minestrone (*vegetable soup*), Cavoli–Cavolfiori-Cime di Rape-Rape (*cabbage*, *cauliflower*, *broccoli*, *green turnips*), Finocchi- Sedano (fennels, celery)
LEGUMES	Ceci - Lenticchie–Fagioli (*chickpeas, lentils, beans*), Piselli (*peas*), Fagiolini (*green beans*), Fave con Verdura (*broad beans with vegetables*)
POTATOES	Patate (*potatoes*)
FRUITS	Arance-Mandarini-Pompelmi (*oranges, tangerines*, *grapefruits*), Pesche (*peaches*), Fichi (*figs*), Albicocche (*apricots*), Uva (*grapes*), Anguria (*watermelon*), Melone giallo (*melon*), Mele-Pere (*apples*, *pears*), Kiwi, Ciliege (cherries), Banane
NUTS	Frutta secca (*nuts*)
GRAINS	*Bread*, Pasta asciutta (*pasta*), Riso o risotti (*rice or risotti*), Pastina o riso in brodo (*pasta or rice in broth*)
OLIVES AND VEGETABLE OIL	Olive da tavola (*olives*), Olio di oliva (*olive oil*)
COOKING EDIBLE FATS	Olio di semi (*seed oil*), Olio di oliva per frittura (*olive oil for frying*), Olio di semi per frittura (*seeds oil for frying*), Olio di oliva per cucinare (*olive oil for cooking*), Olio di semi per cucinare (*seeds oil for cooking*), Burro (*butter*), Margarina (*margarine*), Burro per frittura (*butter for frying*), Margarina per frittura (*margarine for frying*), Burro per cucinare (*butter for cooking*), Margarina per cucinare (*margarine for cooking*)
SWEETS	Caramelle (*sweets*), Cioccolata (*chocolate*), Pasticceria (*pastries*), Biscotti–Paste secche (*cookies, biscuits*)
SUGARY	Zucchero (*sugar*), Frutta sciroppata (*fruit in syrup*)
JUICES	Succhi di frutta (*fruit juice*)
CALORIC DRINKS	Coca Cola–Aranciata–Chinotto (*coke*, *orange juice*, *chinotto*)
READY TO EAT DISH	Pizza, Focaccia (a tipical Apulian bakery product)
COFFEE	Caffè (*coffee*), Caffè d’orzo (*barley coffee*)
WINE	*Wine*
BEER	*Beer*
SPIRITS	Liquore (*liquor*)
WATER	*Water*

**Table A2:** Concordance between the single food items in the questionnaire and the healthy diet indices.

Foods	DASH Diet	MED Diet	MIND Diet
Pasta Asciutta (*Pasta*)	Total Grains	Non Refined Grains	Grains
Riso O Risotti (*Rice Or Risotti*)	Total Grains	Non Refined Grains	Grains
Pastina O Riso In Brodo (*Pasta Or Rice In Broth*)	Total Grains	Non Refined Grains	
Pane (*Bread*)	Total Grains	Non Refined Grains	Grains
Focaccia (*A Tipical Apulian Bakery Product*)	Total Grains	Non Refined Grains	Fast/Fried Food
Pizza	Total Grains	Non Refined Grains	Fast/Fried Food
Vitello (*Calf*)	Meat, Poultry and Fish	Red Meat	Red Meats and Products
Cavallo (*Horse*)	Meat, Poultry and Fish	Red Meat	Red Meats and Products
Maiale (*Pork*)	Meat, Poultry and Fish	Red Meat	Red Meats and Products
Fegato (*Liver*)	Meat, Poultry and Fish	Red Meat	Red Meats and Products
Pollo (*Chicken*)	Meat, Poultry and Fish	Poultry	Poultry
Coniglio (*Rabbit*)	Meat, Poultry and Fish	Poultry	Poultry
Agnello (*Lamb*)	Meat, Poultry and Fish	Red Meat	Red Meats and Products
Salsiccia Fresca (*Fresh Sausages*)	Meat, Poultry and Fish	Red Meat	Red Meats and Products
Prosciutto Crudo (*Raw Ham*)	Meat, Poultry and Fish	Red Meat	Red Meats and Products
Mortadella (A Tipical Italian Cured Meat)	Meat, Poultry and Fish	Red Meat	Red Meats and Products
Salame (*Salami*)	Meat, Poultry and Fish	Red Meat	Red Meats and Products
Uova (*Eggs*)			
Sogliola, Orata, Dentice, Spigola, Cernia (*Sole, Sea Bream, Snapper, Sea Bass, Grouper*)	Meat, Poultry and Fish	Fish	Fish
Merluzzo, Razza, Palombo (*Codfish, Stingray, Dogfish*)	Meat, Poultry and Fish	Fish	Fish
Triglia, Cefalo, Sgombro (*Goatfish, Mullet, Mackerel*)	Meat, Poultry and Fish	Fish	Fish
Polpo, Seppie, Calaoctopus, Sepia, Calamari, Crayfish	Meat, Poultry and Fish	Fish	Fish
Acciughe, Sarde (*Anchovies, Sardines*)	Meat, Poultry and Fish	Fish	Fish
Cozze, Altri Frutti Di Mare (*Mussels*, *Other Seafoods*)	Meat, Poultry and Fish	Fish	Fish
Tonno Sott‘Olio (*Tuna In Oil*)	Meat, Poultry and Fish	Fish	Fish
Latte Intero (*Whole-Fat Milk*)	Dairy	Full Fat Dairy	
Latte Scremato, Latte Parzialmente Scremato (*Skimmed Milk, Semi-Skimmed Milk*)	Dairy		
Yogurt	Dairy	Full Fat Dairy	
Ricotta (*Cottage Cheese*)	Dairy		
Mozzarella (*Mozzarella Cheese*)	Dairy	Full Fat Dairy	
Scamorza, Caciottina Fresca, Stracchino, Fontina (*Semi-Seasoned Italian Cheese*)	Dairy	Full Fat Dairy	Cheese
Bel Paese, Gorgonzola (*Italian Blue Cheese*)	Dairy	Full Fat Dairy	Cheese
Provolone, Caciocavallo (*Seasoned Italian Cheese*)	Dairy	Full Fat Dairy	Cheese
Grana, Parmigiano (*Seasoned Italian Cheese*)	Dairy	Full Fat Dairy	Cheese
Pecorino, Vacchino (*Goat Cheese, Cow Cheese*)	Dairy	Full Fat Dairy	Cheese
Svizzero (*Swiss Cheese*)	Dairy	Full Fat Dairy	Cheese
Formaggino (*Cheese Spread*)	Dairy	Full Fat Dairy	Cheese
Cavoli, Cavolfiori, Cime Di Rape, Rape (*Cabbage, Cauliflower, Broccoli, Green Turnips*)	Vegetables	Vegetables	Green Leafy
Carciofi (*Artichokes*)	Vegetables	Vegetables	Other Vegetables
Zucchine, Melanzane (*Zucchini, Eggplants*)	Vegetables	Vegetables	Other Vegetables
Spinaci (*Spinach*)	Vegetables	Vegetables	Green Leafy
Bietole, Cicorie (*Chard, Chicory*)	Vegetables	Vegetables	Green Leafy
Minestrone (*Vegetable Soup*)	Vegetables	Vegetables	Other Vegetables
Finocchi (*Fennels*)	Vegetables	Vegetables	Other Vegetables
Cetrioli, Cocomeri (*Cucumbers*)	Vegetables	Vegetables	Other Vegetables
Insalata (*Salad*)	Vegetables	Vegetables	Green Leafy
Pomodori (*Tomatoes*)	Vegetables	Vegetables	Other Vegetables
Patate (Patatoes)	Vegetables	Patatoes	Other Vegetables
Carote (Carrots)	Vegetables	Vegetables	Other Vegetables
Peperoni (Peppers)	Vegetables	Vegetables	Other Vegetables
Olive Da Tavola (Olives)		Olive Oil	Olive Oil Primary Oil
Ceci, Lenticchie, Fagioli (*Chickpeas*, *Lentils, Beans*)	Nuts, Seeds and Legumes	Nuts, Seeds and Legumes	Beans
Piselli (*Peas*)	Nuts, Seeds and Legumes	Nuts, Seeds and Legumes	Beans
Fagiolini (*Green Beans*)	Nuts, Seeds and Legumes	Nuts, Seeds and Legumes	Beans
Fave Con Verdure (*Broad Beans With Vegetables*)	Nuts, Seeds and Legumes	Nuts, Seeds and Legumes	Beans
Arance, Mandarini, Pompelmi (*Oranges, Tangerines, Grapefruits*)	Fruits	Fruits	
Pesche (*Peaches*)	Fruits	Fruits	
Fichi (*Figs*)	Fruits	Fruits	
Albicocche (*Apricots*)	Fruits	Fruits	
Uva (*Grapes*)	Fruits	Fruits	
Anguria (*Watermelon*)	Fruits	Fruits	
Melone Giallo (*Melon*)	Fruits	Fruits	
Mele, Pere (*Apples, Pears*)	Fruits	Fruits	
Kiwi	Fruits	Fruits	
Ciliege (*Cherries*)	Fruits	Fruits	
Banana	Fruits	Fruits	
Frutta Secca (*Nuts*)	Nuts, Seeds and Legumes	Nuts, Seeds and Legumes	Nuts
Frutta Sciroppata (*Fruit In Syrup*)	Fruits	Fruits	
Succhi Di Frutta (*Fruit Juice*)	Sweets		Pastries, Sweets
Coca Cola, Aranciata, Chinotto (*Coke, Orange Juice, Chinotto*)	Sweets		Pastries, Sweets
Caramelle (*Sweets*)	Sweets		Pastries, Sweets
Cioccolata (*Chocolate*)	Sweets		Pastries, Sweets
Pasticceria (*Pastries*)	Sweets		Pastries, Sweets
Gelatp (*Ice Cream*)	Sweets		Pastries, Sweets
Biscotti, Paste Secche (*Cookies, Biscuits*)	Sweets		Pastries, Sweets
Vino (*Wine*)		Alcohol	Alcohol/Wine
Birra (*Beer*)		Alcohol	Alcohol/Wine
Liquore (*Liquor*)		Alcohol	Alcohol/Wine
Acqua (*Water*)			
Burro (*Butter*)			Butter, Margarine
Margarina (*Margarine*)			Butter, Margarine
Olio Di Oliva (*Olive Oil*)		Olive Oil	Olive Oil Primary Oil
Olio Di Semi (*Seeds Oil*)			
Burro Per Frittura (*Butter For Frying*)			Fast/Fried Food
Margarina Per Frittura (*Margarine For Frying*)			Fast/Fried Food
Olio Di Oliva Per Frittura (*Olive Oil For Frying*)			Fast/Fried Food
Olio Di Semi Per Frittura (*Seeds Oil For Frying*)			
Burro Per Cucinare (*Butter For Cooking*)			Butter, Margarine
Margarina Per Cucinare (*Margarine For Cooking*)			Butter, Margarine
Olio Di Oliva Per Cucinare (*Olive Oil For Cooking*)			Olive Oil Primary Oil
Olio Di Semi Per Cucinare (*Seeds Oil For Cooking*)			
Caffè (*Coffee*)			
Caffè D‘Orzo (*Barley Coffee*)			
Zucchero (*Sugar*)			
