# Supplementary material for: Traditional Old Dietary Pattern of Castellana Grotte (Apulia) Is Associated with Healthy Outcomes
Source: Nutrients. 2020 Oct 12;12(10):3097. doi: 10.3390/nu12103097 (PMC7600964; doi:10.3390/nu12103097)
Supplement: Supplementary file 1 [file nutrients-12-03097-s001.pdf]

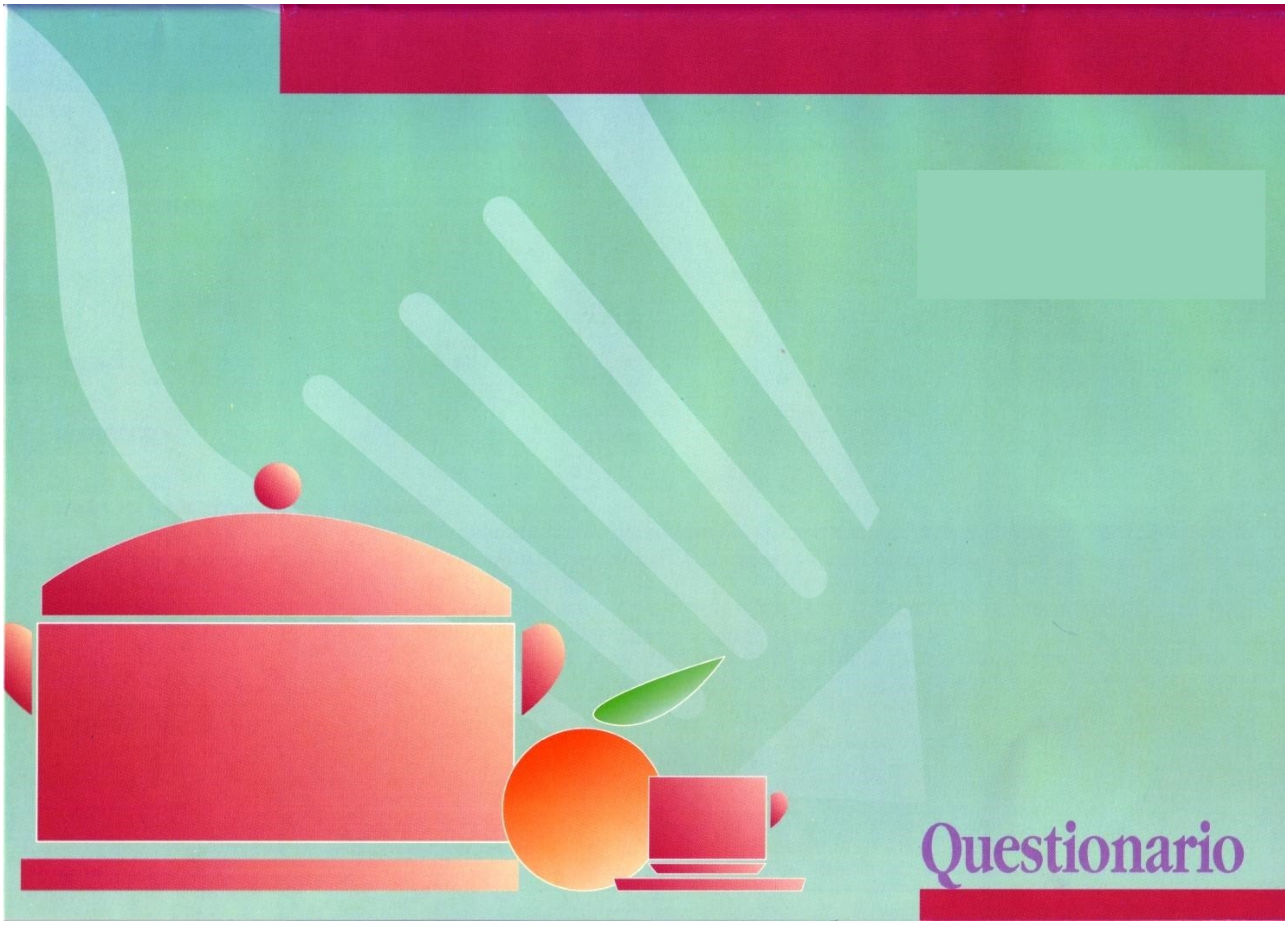

Questionario

Gentile Signore/a,

l'obiettivo del questionario è raccogliere informazioni sulle abitudini di vita della nostra popolazione per studiare se esse possono causare o prevenire alcune importanti malattie, in particolare i tumori.
Ci preme sottolineare che questa ricerca è stata richiesta e finanziata dal Ministero della Sanità.
Ti chiediamo gentilmente di rispondere alle seguenti domande.
Tutte le risposte che cortesemente ci fornirai rimarranno assolutamente riservate.

1 Prima di rispondere alle domande previste prendi visione di tutto il questionario.

2 Rispondi a tutte le domande previste. A seconda dei casi o scrivi la risposta sul rigo oppure indica con una croce (X) il quadratino della risposta prescelta, anche se la risposta è **NO** o **MAI.**

3 Se ti accorgi di aver sbagliato, cancella con la penna la risposta errata e riscrivi quella esatta.

Se hai difficoltà nella compilazione del questionario, per favore non rinunciare! Fatti aiutare da un parente, da un amico o da un vicino di casa. In casi estremi, potresti anche chiedere aiuto al tuo medico di famiglia, se lui è d'accordo ed ha tempo per questo.
Per qualunque informazione, siamo disponibili al numero telefonico seguente: (080)8961394 dalle ore 9 alle 13, tutti i giorni escluso il sabato.

dati anagrafici 1

DATA DI COMPILAZIONE giorno ________ mese ________ anno ________

COGNOME ____________________ **NOME** ____________________

SESSO maschio ☐ femmina ☐

INDIRIZZO Via ____________________ Città ____________ tel. ________

DATA DI NASCITA ____________ **ETA'** ________

COGNOME E NOME DEL TUO MEDICO DI FAMIGLIA ____________________

SE HAI RICEVUTO QUESTO QUESTIONARIO IN OCCASIONE DI UNA VISITA PRESSO L'AMBULATORIO DEL TUO MEDICO CURANTE O DI UN PRELIEVO DI SANGUE PRESSO UN LABORATORIO DI ANALISI, PER FAVORE INDICA IL MOTIVO DEL TUO RICORSO AL SERVIZIO SANITARIO

____

____

____

QUAL È IL TUO TITOLO DI STUDIO?

nessuno ☐ licenza elementare ☐ licenza media ☐ diploma ☐ laurea ☐

CHE ATTIVITA' LAVORATIVA SVOLGI ATTUALMENTE? indica anche se sei pensionato, casalinga o disoccupato

____________________ Da quanti anni? ____________

Se attualmente sei occupato, per quanti giorni lavori alla settimana? ____________

Quante ore lavori al giorno? ____________

Il lavoro che svolgi è soggetto a turni? NO ☐ SI ☐

IN PASSATO HAI SVOLTO UNA ATTIVITA' LAVORATIVA DIVERSA DA QUELLA ATTUALE?

NO ☐ SI ☐ SE SI, specificare quale: ____________________

INDICA SE SEI

celibe o nubile ☐ sposato o convivente ☐ separato o divorziato ☐ vedovo ☐

SOFFRI O HAI MAI SOFFERTO DELLE SEGUENTI MALATTIE? (rispondi NO o SI a tutte le malattie indicate)

DIABETE	NO ☐	SI ☐
PRESSIONE ALTA	NO ☐	SI ☐
GRASSI ELEVATI NEL SANGUE (colesterolo, trigliceridi)	NO ☐	SI ☐
ACIDO URICO ELEVATO NEL SANGUE	NO ☐	SI ☐
EMORRAGIA O TROMBOSI AL CERVELLO	NO ☐	SI ☐
INFARTO DI CUORE	NO ☐	SI ☐
CALCOLI RENALI	NO ☐	SI ☐
BRONCHITE CRONICA	NO ☐	SI ☐
MALATTIE DEL FEGATO (epatite cronica, cirrosi)	NO ☐	SI ☐
CALCOLI ALLA COLECISTI	NO ☐	SI ☐
ULCERA ALLO STOMACO o DUODENALE	NO ☐	SI ☐
POLIPI ALL'INTESTINO	NO ☐	SI ☐
TUMORE	NO ☐	SI ☐

sede del tumore ____________________

ALTRE MALATTIE ____________________

HAI MAI SUBITO OPERAZIONI CHIRURGICHE?

NO ☐ SI ☐

SE SI, per quale malattia?	in che anno?

HAI ALMENO UN PARENTE DI PRIMO GRADO (padre, madre, sorelle, fratelli, figli) CHE HA AVUTO UN TUMORE?

NO ☐ SI ☐ NON SO ☐

SE SI, indica quale dei tuoi parenti di primo grado ha avuto un tumore	sede del tumore

dati generali 4

ATTUALMENTE PRENDI FARMACI PER LA CURA DI QUALCHE MALATTIA?

NO ☐ SI ☐

SE SI, indica il nome delle medicine che prendi

____

____

____

ATTUALMENTE PRENDI VITAMINE?

NO ☐ SI ☐

SE SI, indica il nome delle vitamine che prendi

____

____

____

ATTUALMENTE FUMI SIGARETTE?

NO ☐ SI ☐

SE SI, quante ne fumi al giorno? ______________

Da quanto tempo fumi ? ______________

SE NON FUMI ATTUALMENTE, IN PASSATO FUMAVI?

NO ☐ SI ☐

SE SI, quante ne fumavi al giorno? ______________

Per quanti anni hai fumato? ______________

CON CHE FREQUENZA VAI DI CORPO ABITUALMENTE?

più di 2 volte al giorno ☐ 1 - 2 volte al giorno ☐

1 volta ogni 2 - 3 giorni ☐ meno di 1 volta ogni 3 giorni ☐

FAI USO ABITUALE DI LASSATIVI?

NO ☐ SI ☐

SE SI, quante volte al mese?

____

dati generali 5

QUAL È LA TUA ALTEZZA? cm. ______________

QUAL È IL TUO PESO ATTUALE ? Kg. ______________

UN ANNO FA, RISPETTO AL TUO PESO ATTUALE: avevi lo stesso peso ☐ pesavi di meno ☐ pesavi di più ☐ Se pesavi di meno o di più, quanti Kg. pesavi? ______________

ATTUALMENTE SEGUI UNA DIETA PARTICOLARE? NO ☐ SI ☐

SE SI, quale delle seguenti diete? (rispondi NO o SI a tutte le diete sottoelencate)

dieta dimagrante	NO ☐	SI ☐	SE SI, da quanto tempo? ______
dieta per diabete (pochi zuccheri)	NO ☐	SI ☐	SE SI, da quanto tempo? ______
dieta per grassi elevati nel sangue (pochi grassi)	NO ☐	SI ☐	SE SI, da quanto tempo? ______
dieta per pressione alta (poco sale)	NO ☐	SI ☐	SE SI, da quanto tempo? ______
dieta per acido urico elevato nel sangue (poche proteine)	NO ☐	SI ☐	SE SI, da quanto tempo? ______
altre diete ______________			SE SI, da quanto tempo? ______

HAI PROBLEMI A MASTICARE? NO ☐ SI ☐ SE SI, perché hai problemi di denti? NO ☐ SI ☐ SE SI, da quanti anni? ______

SOLO PER LE DONNE

ATTUALMENTE SEI IN MENOPAUSA? NO ☐ SI ☐

istruzioni

Le successive domande si riferiscono alle tue abitudini alimentari.

1 **Le tue risposte devono fare riferimento alla alimentazione da te seguita nell'anno precedente la compilazione di questo questionario (ovvero l'anno passato!). Se hai cambiato l'alimentazione da meno di un anno (per esempio perché segui una dieta dimagrante o a causa di una malattia) devi riferirti alle tue nuove abitudini alimentari.**

2 **Per ogni cibo o gruppo di cibi elencati ti chiediamo di indicare con una croce (X):**
- quante volte hai mangiato in media quel cibo o gruppo di cibi lo scorso anno (frequenza);
- quanto è grande la quantità che di solito mangi per pasto di quel determinato cibo (porzione).
Indica sempre una sola risposta per la frequenza ed una sola risposta per la porzione.

3 **Per gli alimenti che si mangiano solo in un particolare periodo dell'anno (per esempio l'anguria in estate) la frequenza e la porzione devono riferirsi a quel periodo. Questi cibi sono indicati nel questionario con la frase "quando è il periodo".**

4 **Non è necessario indicare la porzione in questi due casi:**
- quando non è richiesto e non ci sono quindi le caselle apposite (come per esempio per la "focaccia"),
oppure
- se hai risposto MAI alla domanda sulla frequenza.

Nella pagina seguente sono riportati degli esempi.

Compilare il questionario è facile! Stai attento però a non commettere questi errori.

QUANTE VOLTE HAI MANGIATO IN MEDIA I SEGUENTI CIBI LO SCORSO ANNO?

QUANTO È GRANDE LA PORZIONE DI CIBO CHE DI SOLITO MANGI PER PASTO?

	MAI	MENO DI 1 VOLTA AL MESE	1 - 3 VOLTE AL MESE	1 VOLTA ALLA SETT.	2 - 3 VOLTE ALLA SETT.	4 - 6 VOLTE ALLA SETT.	1 VOLTA AL DI'	PIU' DI 1 VOLTA AL DI'	1 - 2 BOCCONCINI o 1/2 treccia	3 - 4 BOCCONCINI o 1 treccia	5 BOCCONCINI o più di 1 treccia
MOZZARELLA		X		X						X	

ERRATO, perché non devi sbarrare la frequenza più di una volta

	MAI	MENO DI 1 VOLTA AL MESE	1 - 3 VOLTE AL MESE	1 VOLTA ALLA SETT.	2 - 3 VOLTE ALLA SETT.	4 - 6 VOLTE ALLA SETT.	1 VOLTA AL DI'	PIU' DI 1 VOLTA AL DI'	1 - 2 BOCCONCINI o 1/2 treccia	3 - 4 BOCCONCINI o 1 treccia	5 BOCCONCINI o più di 1 treccia
MOZZARELLA	X								X		

ERRATO, perché se sbarri la casella MAI e quindi dici di non mangiare mai l'alimento, ovviamente non devi sbarrare la porzione

	MAI	MENO DI 1 VOLTA AL MESE	1 - 3 VOLTE AL MESE	1 VOLTA ALLA SETT.	2 - 3 VOLTE ALLA SETT.	4 - 6 VOLTE ALLA SETT.	1 VOLTA AL DI'	PIU' DI 1 VOLTA AL DI'	1 - 2 BOCCONCINI o 1/2 treccia	3 - 4 BOCCONCINI o 1 treccia	5 BOCCONCINI o più di 1 treccia
MOZZARELLA											

ERRATO, perché se non mangi mai l'alimento non devi lasciare in bianco la risposta ma devi sbarrare la casella MAI

	MAI	MENO DI 1 VOLTA AL MESE	1 - 3 VOLTE AL MESE	1 VOLTA ALLA SETT.	2 - 3 VOLTE ALLA SETT.	4 - 6 VOLTE ALLA SETT.	1 VOLTA AL DI'	PIU' DI 1 VOLTA AL DI'	1 - 2 BOCCONCINI o 1/2 treccia	3 - 4 BOCCONCINI o 1 treccia	5 BOCCONCINI o più di 1 treccia
MOZZARELLA				X							

ERRATO, perché se mangi un alimento devi indicare oltre la porzione anche la frequenza

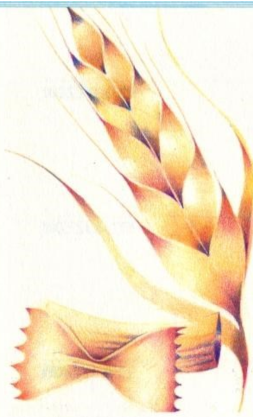

cereali

QUANTE VOLTE HAI MANGIATO IN MEDIA I SEGUENTI CIBI LO SCORSO ANNO?

QUANTO È GRANDE LA PORZIONE DI CIBO CHE DI SOLITO MANGI PER PASTO?

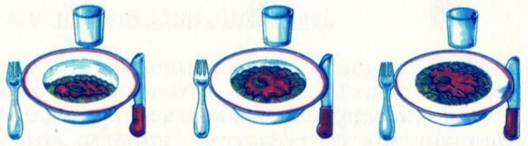

	MAI	MENO DI 1 VOLTA AL MESE	1 - 3 VOLTE AL MESE	1 VOLTA ALLA SETT.	2 - 3 VOLTE ALLA SETT.	4 - 6 VOLTE ALLA SETT.	1 VOLTA AL DI'	PIU' DI 1 VOLTA AL DI'	FONDO DEL PIATTO	PIATTO INTERO	PIATTO ABBONDANTE
PASTA ASCIUTTA											
RISO DA SOLO O RISOTTI											
PASTINA O RISO IN BRODO											

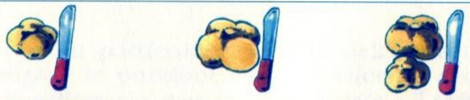

	MAI	MENO DI 1 VOLTA AL MESE	1 - 3 VOLTE AL MESE	1 VOLTA ALLA SETT.	2 - 3 VOLTE ALLA SETT.	4 - 6 VOLTE ALLA SETT.	1 VOLTA AL DI'	PIU' DI 1 VOLTA AL DI'	1 PANINO PICCOLO o 1 fetta di pane	1 PANINO GRANDE o 2 fette di pane	PIU' DI 1 PANINO GRANDE o 3 fette di pane o più
PANE											
FOCACCIA											
PIZZA											

carne

QUANTE VOLTE HAI MANGIATO IN MEDIA I SEGUENTI CIBI LO SCORSO ANNO?

QUANTO È GRANDE LA PORZIONE DI CIBO CHE DI SOLITO MANGI PER PASTO?

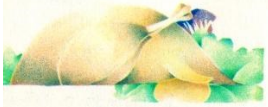

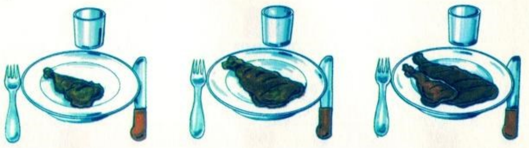

	MAI	MENO DI 1 VOLTA AL MESE	1 - 3 VOLTE AL MESE	1 VOLTA ALLA SETT.	2 - 3 VOLTE ALLA SETT.	4 - 6 VOLTE ALLA SETT.	1 VOLTA AL DI'	PIU' DI 1 VOLTA AL DI'	MENO DEL FONDO DEL PIATTO	FONDO DEL PIATTO	PIU' DEL FONDO DEL PIATTO
VITELLO											
CAVALLO											
MAIALE											
FEGATO											
									MENO DI 1 COSCIA o 1 fettina di petto	1 COSCIA o 2 fettine di petto	PIU' DI 1 COSCIA o più di 2 fettine di petto
POLLO											
									1 PEZZO	2 - 3 PEZZI	PIU' DI 3 PEZZI
CONIGLIO											
									1 PEZZO	2 - 3 PEZZI	PIU' DI 3 PEZZI
AGNELLO											
									1 - 2 PEZZI	3 - 4 PEZZI	PIU' DI 4 PEZZI
SALSICCIA FRESCA											

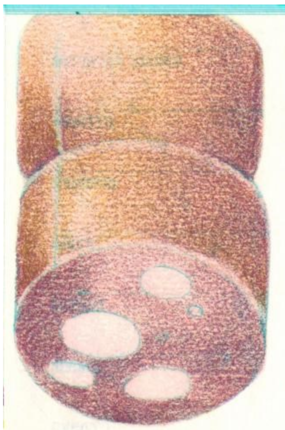

carne

QUANTE VOLTE HAI MANGIATO IN MEDIA I SEGUENTI CIBI LO SCORSO ANNO?

QUANTO È GRANDE LA PORZIONE DI CIBO CHE DI SOLITO MANGI PER PASTO?

	MAI	MENO DI 1 VOLTA AL MESE	1 - 3 VOLTE AL MESE	1 VOLTA ALLA SETT.	2 - 3 VOLTE ALLA SETT.	4 - 6 VOLTE ALLA SETT.	1 VOLTA AL DI'	PIU' DI 1 VOLTA AL DI'
PROSCIUTTO CRUDO								
MORTADELLA - PROSCIUTTO COTTO								
SALAME								
UOVA								

	Porzione		
PROSCIUTTO CRUDO	1 FETTA	2 FETTE	3 o PIU' FETTE
MORTADELLA - PROSCIUTTO COTTO	1 FETTA	2 FETTE	3 o PIU' FETTE
SALAME	1 - 4 FETTE	5 - 10 FETTE	PIU' DI 10 FETTE
UOVA	1 UOVO	2 UOVA o PIU'	

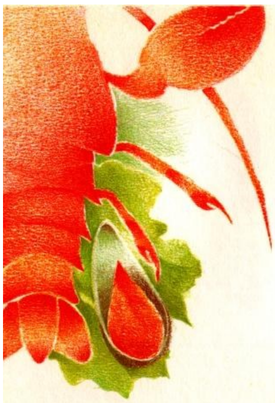

pesce

QUANTE VOLTE HAI MANGIATO IN MEDIA I SEGUENTI CIBI LO SCORSO ANNO?

QUANTO È GRANDE LA PORZIONE DI CIBO CHE DI SOLITO MANGI PER PASTO?

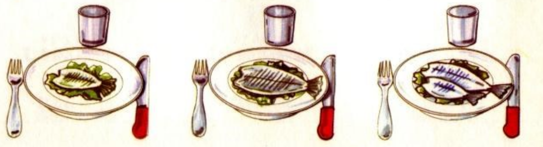

	MAI	MENO DI 1 VOLTA AL MESE	1 - 3 VOLTE AL MESE	1 VOLTA ALLA SETT.	2 - 3 VOLTE ALLA SETT.	4 - 6 VOLTE ALLA SETT.	1 VOLTA AL DI'	PIU' DI 1 VOLTA AL DI'
SOGLIOLA - ORATA - DENTICE - SPIGOLA - CERNIA								
MERLUZZO - RAZZA - PALOMBO								
TRIGLIA - CEFALO - SGOMBRO								
POLPO - SEPPIE - CALAMARI - GAMBERI								
ACCIUGHE - SARDE								
COZZE - ALTRI FRUTTI DI MARE								
TONNO SOTT'OLIO								

	Porzione		
SOGLIOLA - ORATA - DENTICE - SPIGOLA - CERNIA	MENO DEL FONDO DEL PIATTO	FONDO DEL PIATTO	PIU' DEL FONDO DEL PIATTO
MERLUZZO - RAZZA - PALOMBO			
TRIGLIA - CEFALO - SGOMBRO			
POLPO - SEPPIE - CALAMARI - GAMBERI			
ACCIUGHE - SARDE			
COZZE - ALTRI FRUTTI DI MARE			
TONNO SOTT'OLIO	MENO DI 1 SCATOLA PICCOLA	1 SCATOLA PICCOLA (80 gr)	PIU' DI 1 SCATOLA PICCOLA

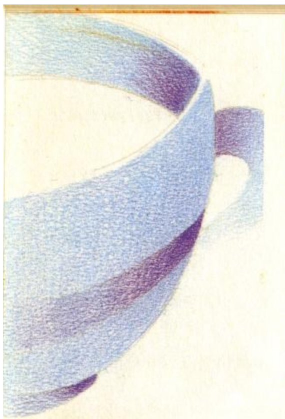

latte e derivati del latte

QUANTE VOLTE HAI MANGIATO IN MEDIA I SEGUENTI CIBI LO SCORSO ANNO?

QUANTO È GRANDE LA PORZIONE DI CIBO CHE DI SOLITO MANGI PER PASTO?

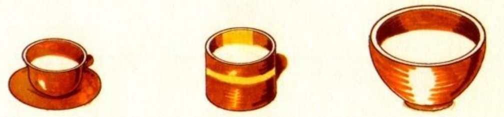

	MAI	MENO DI 1 VOLTA AL MESE	1 - 3 VOLTE AL MESE	1 VOLTA ALLA SETT.	2 - 3 VOLTE ALLA SETT.	4 - 6 VOLTE ALLA SETT.	1 VOLTA AL DI'	PIU' DI 1 VOLTA AL DI'	1 TAZZA DA CAPPUCCINO (circa 100 cc)	1 TAZZA DA LATTE (circa 200 cc)	1 TAZZONE DA LATTE (circa 300 cc)
LATTE INTERO (da solo o con caffé)											
LATTE SCREMATO - PARZIALMENTE SCREMATO (da solo o con caffé)											
YOGURT											
RICOTTA									MENO DI 1/4 DI CESTELLO	1/4 DI CESTELLO	PIU' DI 1/4 DI CESTELLO
MOZZARELLA									1 - 2 BOCCONCINI o 1/2 treccia	3 - 4 BOCCONCINI o 1 treccia	5 BOCCONCINI o più di 1 treccia

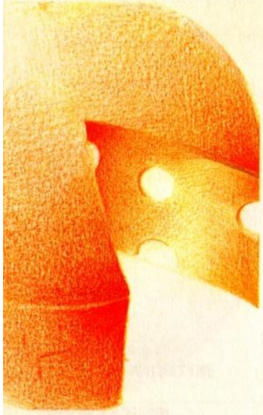

latte e derivati del latte

QUANTE VOLTE HAI MANGIATO IN MEDIA I SEGUENTI CIBI LO SCORSO ANNO?

QUANTO È GRANDE LA PORZIONE DI CIBO CHE DI SOLITO MANGI PER PASTO?

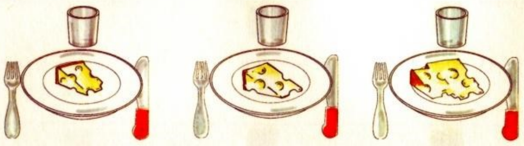

	MAI	MENO DI 1 VOLTA AL MESE	1 - 3 VOLTE AL MESE	1 VOLTA ALLA SETT.	2 - 3 VOLTE ALLA SETT.	4 - 6 VOLTE ALLA SETT.	1 VOLTA AL DI'	PIU' DI 1 VOLTA AL DI'	MENO DI 1 FETTA	1 FETTA	2 o PIU' FETTE
SCAMORZA											
CACIOTTINA FRESCA - STRACCHINO - FONTINA											
BEL PAESE - GORGONZOLA											
PROVOLONE - CACIOCAVALLO											
GRANA - PARMIGIANO											
PECORINO - VACCHINO											
SVIZZERO											
FORMAGGINO									1 FORMAGGINO	2 FORMAGGINI	3 o PIU' FORMAGGINI

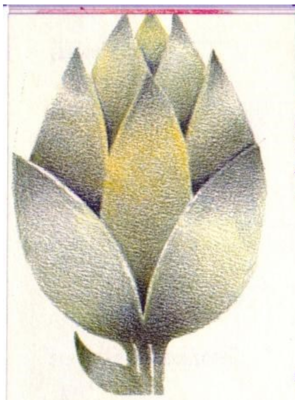

verdura e ortaggi

QUANTE VOLTE HAI MANGIATO IN MEDIA I SEGUENTI CIBI LO SCORSO ANNO?

QUANTO È GRANDE LA PORZIONE DI CIBO CHE DI SOLITO MANGI PER PASTO?

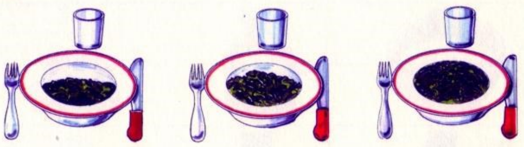

	MAI	MENO DI 1 VOLTA AL MESE	1 - 3 VOLTE AL MESE	1 VOLTA ALLA SETT.	2 - 3 VOLTE ALLA SETT.	4 - 6 VOLTE ALLA SETT.	1 VOLTA AL DI'	PIU' DI 1 VOLTA AL DI'	FONDO DEL PIATTO	PIATTO INTERO	PIATTO ABBONDANTE
CAVOLI - CAVOLFIORI - CIME DI RAPE - RAPE quando è il periodo											
CARCIOFI quando è il periodo											
ZUCCHINE - MELANZANE quando è il periodo											
SPINACI											
BIETOLE - CICORIE											
MINESTRONE											
FINOCCHI - SEDANO quando è il periodo									META'	1 INTERO	1 e 1/2 o PIU'
CETRIOLI - COCOMERI quando è il periodo									META'	1 INTERO	1 e 1/2 o PIU'

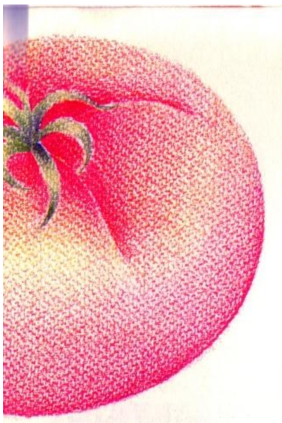

verdura e ortaggi

QUANTE VOLTE HAI MANGIATO IN MEDIA I SEGUENTI CIBI LO SCORSO ANNO?

QUANTO È GRANDE LA PORZIONE DI CIBO CHE DI SOLITO MANGI PER PASTO?

	MAI	MENO DI 1 VOLTA AL MESE	1 - 3 VOLTE AL MESE	1 VOLTA ALLA SETT.	2 - 3 VOLTE ALLA SETT.	4 - 6 VOLTE ALLA SETT.	1 VOLTA AL DI'	PIU' DI 1 VOLTA AL DI'	FONDO DEL PIATTO	PIATTO INTERO	PIATTO ABBONDANTE
INSALATA											
POMODORI (da soli o con l'insalata)											
PATATE											
CAROTE											
PEPERONI											
OLIVE DA TAVOLA											

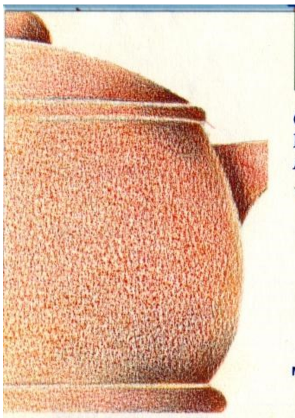

legumi

QUANTE VOLTE HAI MANGIATO IN MEDIA I SEGUENTI CIBI LO SCORSO ANNO?

QUANTO È GRANDE LA PORZIONE DI CIBO CHE DI SOLITO MANGI PER PASTO?

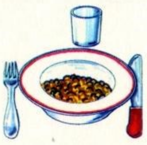

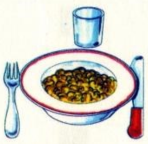

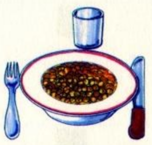

	MAI	MENO DI 1 VOLTA AL MESE	1 - 3 VOLTE AL MESE	1 VOLTA ALLA SETT.	2 - 3 VOLTE ALLA SETT.	4 - 6 VOLTE ALLA SETT.	1 VOLTA AL DI'	PIU' DI 1 VOLTA AL DI'	FONDO DEL PIATTO	PIATTO INTERO	PIATTO ABBONDANTE
**CECI - LENTICCHIE - FAGIOLI** da soli o con pasta o riso											
**PISELLI** da soli o con pasta o riso											
**FAGIOLINI** da soli o con pasta quando è il periodo											
**FAVE CON VERDURA**											

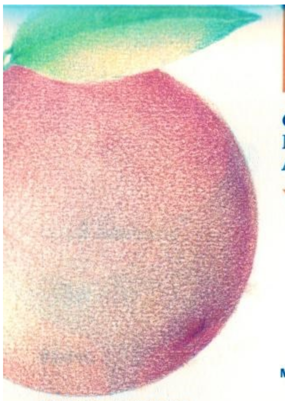

frutta

QUANTE VOLTE HAI MANGIATO IN MEDIA I SEGUENTI CIBI LO SCORSO ANNO?

QUANTO È GRANDE LA PORZIONE DI CIBO CHE DI SOLITO MANGI PER PASTO?

	MAI	MENO DI 1 VOLTA AL MESE	1 - 3 VOLTE AL MESE	1 VOLTA ALLA SETT.	2 - 3 VOLTE ALLA SETT.	4 - 6 VOLTE ALLA SETT.	1 VOLTA AL DI'	PIU' DI 1 VOLTA AL DI'			
**ARANCE - MANDARINI - POMPELMI** quando è il periodo									MENO DI 1 FRUTTO	1 FRUTTO	2 o PIU' FRUTTI
**PESCHE** quando è il periodo											
**FICHI** quando è il periodo									1 - 3 FRUTTI	4 - 5 FRUTTI	6 o PIU' FRUTTI
**ALBICOCCHE** quando è il periodo									1 FRUTTO	2 - 3 FRUTTI	4 o PIU' FRUTTI
**UVA** quando è il periodo									MENO DI 1 GRAPPOLO	1 GRAPPOLO	2 o PIU' GRAPPOLI
**ANGURIA (melone rosso)** quando è il periodo									MENO DI 1 FETTA	1 FETTA	2 o PIU' FETTE
**MELONE GIALLO** quando è il periodo											
**MELE - PERE**									MENO DI 1 FRUTTO	1 FRUTTO	2 o PIU' FRUTTI

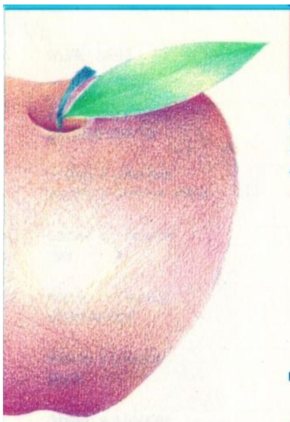

frutta

QUANTE VOLTE HAI MANGIATO IN MEDIA I SEGUENTI CIBI LO SCORSO ANNO?

	MAI	MENO DI 1 VOLTA AL MESE	1 - 3 VOLTE AL MESE	1 VOLTA ALLA SETT.	2 - 3 VOLTE ALLA SETT.	4 - 6 VOLTE ALLA SETT.	1 VOLTA AL DI'	PIU' DI 1 VOLTA AL DI'
KIWI								
CILIEGE quando è il periodo								
BANANE								
FRUTTA SECCA								
FRUTTA SCIROPPATA								

QUANTO È GRANDE LA PORZIONE DI CIBO CHE DI SOLITO MANGI PER PASTO?

KIWI	MENO DI 1 FRUTTO	1 FRUTTO	2 o PIU' FRUTTI
CILIEGE quando è il periodo			
BANANE	MENO DI 1 FRUTTO	1 FRUTTO	2 o PIU' FRUTTI
FRUTTA SECCA	MENO DI 10 FRUTTI	10 o PIU' FRUTTI	
FRUTTA SCIROPPATA	1 PEZZO	2 - 3 PEZZI	4 o PIU' PEZZI

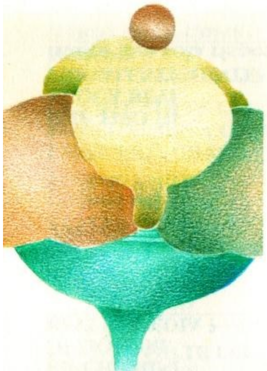

alimenti vari

QUANTE VOLTE HAI MANGIATO IN MEDIA I SEGUENTI CIBI LO SCORSO ANNO?

	MAI	MENO DI 1 VOLTA AL MESE	1 - 3 VOLTE AL MESE	1 VOLTA ALLA SETT.	2 - 3 VOLTE ALLA SETT.	4 - 6 VOLTE ALLA SETT.	1 VOLTA AL DI'	PIU' DI 1 VOLTA AL DI'
SUCCHI DI FRUTTA								
COCA COLA - ARANCIATA - CHINOTTO nel periodo estivo								
COCA COLA - ARANCIATA - CHINOTTO escluso nel periodo estivo								
CARAMELLE								
CIOCCOLATA								
PASTICCERIA torte o pasticcini								
GELATO solo nel periodo estivo								
BISCOTTI - PASTE SECCHE								

QUANTO È GRANDE LA PORZIONE DI CIBO CHE DI SOLITO MANGI PER PASTO?

SUCCHI DI FRUTTA	1 BOTTIGLIETTA	2 o PIU' BOTTIGLIETTE	
COCA COLA - ARANCIATA - CHINOTTO nel periodo estivo	1/2 BICCHIERE	1 BICCHIERE	2 o PIU' BICCHIERI
COCA COLA - ARANCIATA - CHINOTTO escluso nel periodo estivo	(no label printed)	(no label printed)	(no label printed)
CARAMELLE	1 - 2 CARAMELLE	3 - 4 CARAMELLE	5 o PIU' CARAMELLE
CIOCCOLATA	1 CIOCCOLATINO o 1/2 tavoletta	2 - 3 CIOCCOLATINI o 1 tavoletta	4 CIOCCOLATINI o 2 o più tavolette
PASTICCERIA torte o pasticcini	1 PEZZO	2 o PIU' PEZZI	
GELATO solo nel periodo estivo			
BISCOTTI - PASTE SECCHE	1 - 3	4 - 6	7 o PIU'

acqua e alcolici

**QUANTO VINO BEVI IN MEDIA?**	NON NE BEVO O NE BEVO SALTUARIAMENTE	MEZZO BICCHIERE AL GIORNO	1 BICCHIERE AL GIORNO	2 - 3 BICCHIERI AL GIORNO	4 - 5 BICCHIERI AL GIORNO	PIU' DI 5 BICCHIERI AL GIORNO
**QUANTA BIRRA BEVI IN MEDIA ? escluso nel periodo estivo**	NON NE BEVO O NE BEVO SALTUARIAMENTE	CIRCA UNA BOTTIGLIA PICCOLA DA UN TERZO AL GIORNO	CIRCA UNA BOTTIGLIA GRANDE DA DUE TERZI AL GIORNO	PIU ' DI UNA BOTTIGLIA GRANDE AL GIORNO		
**QUANTI BICCHIERINI DI LIQUORE BEVI IN MEDIA ?**	NON NE BEVO O NE BEVO MENO DI UNO ALLA SETTIMANA	1 - 2 ALLA SETTIMANA	3 - 5 ALLA SETTIMANA	6 - 7 ALLA SETTIMANA	1 - 3 AL GIORNO	PIU' DI 3 AL GIORNO
**QUANTA ACQUA BEVI IN MEDIA? escluso nel periodo estivo**	NON NE BEVO O NE BEVO SALTUARIAMENTE	1 - 2 BICCHIERI AL GIORNO	3 - 4 BICCHIERI AL GIORNO	CIRCA UN LITRO AL GIORNO	UN LITRO E MEZZO AL GIORNO	CIRCA 2 LITRI AL GIORNO
**CHE TIPO DI ACQUA BEVI PREVALENTEMENTE? indica una sola risposta**	DI ACQUEDOTTO		DI POZZO		MINERALE	

olio e grassi

dai una risposta a tutti i condimenti elencati

**QUANTE VOLTE USI PER CONDIRE A CRUDO I SEGUENTI GRASSI ?**	MAI	MENO DI 1 VOLTA A SETTIMANA	1 VOLTA A SETTIMANA	2 - 3 VOLTE A SETTIMANA	4 - 6 VOLTE A SETTIMANA	1 O PIU' VOLTE AL GIORNO
BURRO						
MARGARINA						
OLIO DI OLIVA						
OLIO DI SEMI						

**QUANTE VOLTE USI PER FRIGGERE I SEGUENTI GRASSI ?**	MAI	MENO DI 1 VOLTA A SETTIMANA	1 VOLTA A SETTIMANA	2 - 3 VOLTE A SETTIMANA	4 - 6 VOLTE A SETTIMANA	1 O PIU' VOLTE AL GIORNO
BURRO						
MARGARINA						
OLIO DI OLIVA						
OLIO DI SEMI						

**QUANTE VOLTE USI PER CUCINARE I SEGUENTI GRASSI (escluso la frittura)?**	MAI	MENO DI 1 VOLTA A SETTIMANA	1 VOLTA A SETTIMANA	2 - 3 VOLTE A SETTIMANA	4 - 6 VOLTE A SETTIMANA	1 O PIU' VOLTE AL GIORNO
BURRO						
MARGARINA						
OLIO DI OLIVA						
OLIO DI SEMI						

QUANTI LITRI DI OLIO DI OLIVA SI CONSUMANO IN CASA TUA IN UN ANNO? ____________

DI SOLITO, IN UN GIORNO FERIALE IN QUANTI SIETE A TAVOLA COMPRESO TE? ____________

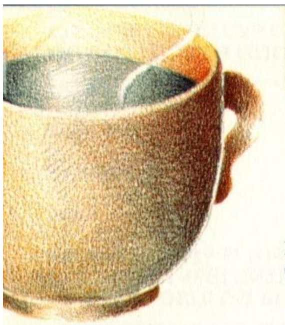

caffè, zucchero e sale

BEVI CAFFÉ?
(compreso quello aggiunto al latte) — NO ☐ SI ☐ — **SE SI**, quante tazzine di caffè bevi in media al giorno? ________

BEVI CAFFÉ D'ORZO? — NO ☐ SI ☐ — **SE SI**, quante tazze di caffè d'orzo bevi in media al giorno? ________

FAI USO DI ZUCCHERO? — NO ☐ SI ☐ — **SE SI**, quanti cucchiaini di zucchero consumi durante la giornata? ________

Rispondi a questa domanda solo se ti capita almeno una volta l'anno di mangiare fuori casa (da parenti, da amici, al ristorante):
COME TROVI IL CIBO PREPARATO FUORI CASA?

troppo salato ☐ senza sale ☐ salato normalmente ☐

attività fisica

Le seguenti domande si riferiscono all'anno precedente la compilazione di questo questionario e riguardano le attività che sei solito svolgere durante una tua tipica giornata feriale, quindi escluse le domeniche e i giorni festivi.

A CHE ORA VAI A LETTO DI SOLITO? ________

A CHE ORA TI ALZI DI SOLITO? ________

FAI UN RIPOSO DURANTE IL POMERIGGIO? — NO ☐ SI ☐ — **SE SI**, quanto tempo dura in media il tuo riposo pomeridiano? ORE ________ MINUTI ________

DURANTE LA TUA GIORNATA TI DEDICHI ALLA CUCINA O LAVI I PIATTI? — NO ☐ SI ☐ — **SE SI**, quanto tempo al giorno dedichi in media alla cucina o a lavare i piatti? ORE ________ MINUTI ________

DURANTE LA TUA GIORNATA TI DEDICHI ALLA PULIZIA DELLA CASA?
spolveri, lavi indumenti, pulisci i vetri, lavi e lucidi i pavimenti, ecc. — NO ☐ SI ☐ — **SE SI**, quanto tempo dedichi in media ai lavori di pulizia della casa? ORE ________ MINUTI ________

NEL TUO TEMPO LIBERO, QUINDI NON PER LAVORO, VAI IN CAMPAGNA O IN GIARDINO A COLTIVARE LA TERRA?
zappi, tagli erbacce, pianti verdure, poti alberi, raccogli frutta, ecc. — NO ☐ SI ☐ — **SE SI**, quanto tempo dedichi in media durante una tua giornata feriale a coltivare la terra o a fare giardinaggio? ORE ________ MINUTI ________

Le seguenti domande si riferiscono all'anno precedente la compilazione di questo questionario e riguardano le attività che sei solito svolgere durante una tua tipica giornata feriale, quindi escluse le domeniche e i giorni festivi.

DURANTE LA TUA GIORNATA FERIALE ESCI DI CASA PER PASSEGGIARE, FARE COMPERE O CAMMINARE IN GENERE?

NO ☐ SI ☐

SE SI, per quanto tempo in media al giorno sei fuori casa per passeggiare, fare compere o camminare in genere? ORE ______ MINUTI ______

HAI UN PASSATEMPO? escluso le passeggiate

NO ☐ SI ☐

SE SI, quale? ______

quante volte alla settimana ti dedichi al tuo passatempo? ______

quanto tempo in media dedichi ogni volta al tuo passatempo? ORE ______ MINUTI ______

USI DI SOLITO LA BICICLETTA PER SPOSTARTI O FARE PASSEGGIATE?

NO ☐ SI ☐

SE SI, quante volte alla settimana? ______

quanto tempo in media vai ogni volta in bicicletta? ORE ______ MINUTI ______

FAI ESERCIZI DI GINNASTICA?

NO ☐ SI ☐

SE SI, quante volte durante la settimana fai esercizi di ginnastica? ______

quanto tempo dedichi in media ogni volta ai tuoi esercizi di ginnastica? ORE ______ MINUTI ______

Questionario a cura del:

LABORATORIO
DI EPIDEMIOLOGIA
E BIOSTATISTICA

I.R.C.C.S "S. De Bellis"
Ospedale Gastroenterologico

Via Valente, 4 - 70013 Castellana (Ba) - Tel. (080) 8961394
